# Multi-omics integration reveals tissue-specific biosynthesis of sesquiterpenoids in the medicinal plant *Eupatorium lindleyanum*

**DOI:** 10.1371/journal.pone.0359566

**Published:** 2026-10-01

**Authors:** Yingzhe Wang, Pan Jiang, Jiaqiu Yuan, Jinghan Wu, Yue Zhang, Kun Guo

**Affiliations:** 1 School of Pharmacy, Jiangsu Food and Pharmaceutical Science College, Huaian, China; 2 College of Traditional Chinese Medicine, Jilin Agricultural Science and Technology College, Jilin City, China; 3 School of Pharmaceutical Engineering, Jiangsu Food and Pharmaceutical Science College, Huaian, China; Anhui University of Chinese Medicine, CHINA

## Abstract

**Background:**

*Eupatorium lindleyanum* is a medicinal plant rich in bioactive sesquiterpenoids, yet the genetic and regulatory basis underlying their biosynthesis remains completely unexplored. In particular, the marked tissue-specific accumulation of these compounds—abundant in aerial organs but scarce in roots—lacks a molecular explanation.

**Results:**

Here, we integrated UPLC-MS/MS-based metabolomics with RNA-Seq transcriptomics to comprehensively profile sesquiterpenoid metabolites and their associated gene expression signatures across root, stem, leaf, and flower tissues of *Eupatorium lindleyanum*. A total of 321 sesquiterpenoids were identified, exhibiting distinct tissue-specific accumulation patterns with highest abundance in flower, followed by leaf. Comparative analysis revealed 299 differentially accumulated sesquiterpenoid metabolites (DSMs), which were clustered into three major groups with preferential accumulation in leaf (Cluster 1), flower (Cluster 2), and root (Cluster 3). Transcriptome sequencing generated 99.43 Gb of clean data, yielding 147743 unigenes. Integration of metabolomic and transcriptomic datasets screened 20 structural genes (88 unigenes) involved in sesquiterpenoid biosynthesis, including MVA pathway genes (*ACAT*, *HMGCS*, *HMGCR*, *MVK*, *PMK*, *MVD*), MEP pathway genes (*dxs*, *dxr*, *ispD*, *ispE*, *ispF*, *ispG*, *ispH*), and downstream modification genes (*FDPS*, *IDI*, *GAS*, *GDS*, *NES1*, *GAO*, *FLDH*). Weighted gene co-expression network analysis (WGCNA) further prioritized hub genes within modules significantly correlated with key sesquiterpenoids. Additionally, five transcription factor families (*AP2/ERF*, *NAC*, *WRKY*, *MYB*, and *bHLH*) were identified as potential regulators based on strong correlations with DSMs accumulation.

**Conclusions:**

This study provides the first comprehensive view of tissue-specific sesquiterpenoid biosynthesis in *Eupatorium lindleyanum*, establishing a prioritized set of candidate genes and regulators for functional validation. Our findings offer a foundational resource for metabolic engineering and molecular breeding aimed at enhancing the production of pharmacologically active sesquiterpenoids in this medicinal species.

## 1. Background

*Eupatorium lindleyanum*, a perennial herbaceous plant belonging to the Asteraceae family, is indigenous to East Asia [1]. In China, it is particularly abundant in the Xuyi County area of Jiangsu Province [2]. According to the theories of Traditional Chinese Medicine (TCM), this plant serves as the botanical origin of the crude drug “Yema Zhui,” which is traditionally ascribed with therapeutic effects including resolving phlegm, alleviating cough, and relieving dyspnea [3]. Clinically, it has been employed in the management of chronic tracheitis and bronchitis [4]. Pharmacognostical and phytochemical investigations into the medicinal properties of *Eupatorium lindleyanum* commenced in the 1970s [5]. Through extensive research spanning several decades, over one hundred distinct compounds have been isolated and characterized from this species. These isolates encompass diverse classes of secondary metabolites, including sesquiterpenoids, flavonoids, and alkaloids [6]. Among these, the sesquiterpenoids constitute the class exhibiting the most potent bioactivities. Previous studies have successfully identified numerous bioactive sesquiterpenoids from *Eupatorium lindleyanum*, such as eupalinolides and eupalinilides, among others. These compounds have demonstrated significant pharmacological effects, including the prevention of lung injury, reduction of blood lipid levels, antitumor activity, attenuation of inflammatory responses, and free radical scavenging capacity ^[^7–9^]^. Collectively, these findings provide a scientific basis that substantiates the plant’s traditional medicinal applications. The significant pharmaceutical potential of these bioactive sesquiterpenoids positions them as key targets for metabolic engineering and as lead compounds for further development [10].

Despite progress in other species, how sesquiterpenoids are biosynthesized in *Eupatorium lindleyanum* remains largely unknown. Although the general framework of sesquiterpenoid biosynthesis in plants has been well established, recent investigations in the Asteraceae family have uncovered unique mechanistic innovations and variations within this pathway [11]. For instance, a functionally distinct cytochrome P450 enzyme (*CYP71DD1*) identified in *Xanthium strumarium* catalyzes the skeletal rearrangement of the germacrane backbone into a xanthanolide-type framework, a critical step in the formation of 12,8-cis-lactones [12]. Furthermore, the sesquiterpene synthase *IlTPS1*, cloned from *Inula linearifolia*, was found to direct the biosynthesis of rare bridged tricyclic presilphiperfolane-type sesquiterpenoids, with its products exhibiting significant antifungal activity [13]. Similarly, comparative genomic analyses of *Silybum marianum* and *Carthamus tinctorius* have revealed the presence of biosynthetic gene clusters responsible for caryophyllene-type sesquiterpenoids, whose formation is closely associated with whole-genome duplication and tandem duplication events [14]. These findings highlight the unique and diverse nature of sesquiterpenoid biosynthesis in Asteraceae, characteristics that are not fully captured by the traditional view of a highly conserved universal metabolic pathway. Despite this progress in related species, the specific genetic and regulatory basis for sesquiterpenoid biosynthesis in *Eupatorium lindleyanum* remains largely unexplored. To date, none of the genes encoding key enzymes in this pathway have been functionally characterized, and the tissue-specific regulatory mechanisms governing their expression are yet to be investigated. Addressing this knowledge gap is essential, as it would not only clarify whether *Eupatorium lindleyanum* employs unique regulatory strategies compared to other Asteraceae plants but also unlock the significant medicinal potential of its bioactive sesquiterpenoids. Elucidating this pathway holds substantial application value: it would enable the metabolic engineering of key sesquiterpenoid compounds, support the breeding of high-yielding *Eupatorium lindleyanum* varieties, and inform cultivation practices to enhance the accumulation of these bioactive compounds. Ultimately, a comprehensive understanding of this pathway will contribute to the development of more effective herbal medicines and strengthen the pharmacological application of *Eupatorium lindleyanum* [15].

Integrated metabolomic and transcriptomic analysis has emerged as a pivotal approach for elucidating the biosynthesis and regulation of plant secondary metabolites. This strategy employs LC-MS/MS to comprehensively profile metabolite accumulation patterns, coupled with RNA-seq to identify differentially expressed genes [16]. Through correlation analysis and pathway enrichment, it enables the construction of gene–metabolite regulatory networks and the identification of key regulatory nodes in critical metabolic pathways [17]. This integrated approach has been successfully applied in diverse plant species, including *Cyclocarya paliurus* [18], *Camphora officinarum* [19], *Opisthopappus taihangensis* [20], and *Bidens alba* [21], providing support for deciphering important secondary metabolic pathways such as terpenoids biosynthesis and for mining key functional genes.

In this study, we collected samples from four distinct tissues—root, stem, leaf, and flower—of *Eupatorium lindleyanum* and performed comprehensive metabolomic profiling via UPLC-MS/MS alongside RNA-seq-based transcriptomic sequencing. Using integrated metabolomic and transcriptomic analyses, we systematically investigated the tissue-specific accumulation patterns of sesquiterpenoids and identified key structural genes and transcription factors involved in their biosynthesis. Through weighted gene co-expression network analysis (WGCNA) and correlation analysis between differentially accumulated sesquiterpenoid metabolites and differentially expressed genes, we constructed a sesquiterpenoid biosynthetic regulatory network in *Eupatorium lindleyanum.* These findings provide a valuable resource for understanding the molecular mechanisms governing sesquiterpenoid biosynthesis and offer potential targets for genetic engineering and breeding programs aimed at enhancing the production of bioactive compounds in this medicinal plant.

## 2. Materials and methods

### 2.1. Plant materials

On 14 September 2025, the *Eupatorium lindleyanum* specimens were harvested from a cultivated plantation in Xuyi County, Jiangsu province, China (32°55′37.831″N, 118°25′46.878″E), all of which were 17-month-old plants at the full-flowering stage. Taxonomic identification was performed by Professor Meng Zhu (Jiangsu Food and Pharmaceutical Science College) following the morphological descriptors in the Flora of China. Supplementary Figure 1 in S1 File provides a detailed view of the plant tissues. A voucher specimen (accession no. NAS00718179) has been archived in the publicly accessible herbarium of the Nanjing Botanical Garden, Chinese Academy of Sciences, and is available for examination upon formal request to the curator (bgs@cnbg.net). For each tissue type, three biological replicates were prepared by separately collecting samples from three distinct individual plants, ensuring that each replicate was independent and representative of the tissue type [22]. After collection, specimens were rinsed with ultrapure water, snap-frozen in liquid nitrogen within 1 minute, and maintained at −80°C pending subsequent analyses [23].

### 2.2. Plant sample pretreatment and metabolite extraction procedures

A previously described extraction protocol was used with minor modifications [24]. *Eupatorium lindleyanum* tissues were ground to a fine powder using a ball mill (30 Hz, 1.5 min; Scientz-100F, Ningbo, China). Each 50 mg of powder was mixed with 1.2 mL of 70% methanol pre-chilled to −20°C. This solvent-to-sample ratio was chosen to ensure full submersion of the tissue and efficient release of metabolites across a wide polarity range. The mixture underwent six rounds of vortexing (30 seconds each) at 30-minute intervals. After centrifugation, the resulting supernatant was passed through a 0.22 μm membrane filter before UPLC‒MS/MS measurement. Given that many sesquiterpenoids exist as thermally labile lactones or polar glycosides that are poorly amenable to gas chromatography, the UPLC‒MS/MS platform was selected to enable comprehensive detection without derivatization or thermal degradation.

### 2.3. UPLC‒MS/MS conditions for metabolite profiling

UPLC-MS/MS analysis was conducted using an ExionLC™ AD chromatograph coupled to an ESI-QTRAP mass spectrometer (https://sciex.com.cn/). A reversed-phase separation was performed on an Agilent SB-C18 column (2.1 × 100 mm, 1.8 μm particle size). The mobile phase consisted of (A) 0.1% formic acid in water and (B) 0.1% formic acid in acetonitrile. The elution gradient was programmed as follows: 0–9 min, 5%–95% B; 9–10 min, hold at 95% B; 10–11.1 min, return to 5% B; and 11.1–14 min, re-equilibration with 5% B. Operational parameters included a flow rate of 0.35 mL/min, a column oven temperature of 40°C, and an injection volume of 2 μL. The column outflow was directed into the mass spectrometer for data acquisition [25].

ESI source settings were configured as follows: temperature, 500°C; ion spray voltage, + 5500 V (positive) and −4500 V (negative); nebulizer gas (GSI), heater gas (GSII), and curtain gas (CUR) at 50, 60, and 25 psi, respectively; with collision-activated dissociation (CAD) set to high. Mass spectral data were collected in multiple reaction monitoring (MRM) mode using nitrogen as the collision gas at a medium setting [26].

### 2.4. Qualitative and quantitative analysis of metabolites

Metabolite identification, quantification, and multivariate analysis were performed as described previously [2]. Briefly, identification used the MetWare database, quantification used triple quadrupole MS in MRM mode, and data were processed with Analyst 1.6.3. PCA and OPLS-DA were conducted in R (prcomp) and MetaboAnalyst 6.0, respectively. For the OPLS-DA models, the six pairwise comparison groups (Flower vs Leaf, Flower vs Root, Flower vs Stem, Leaf vs Root, Leaf vs Stem, and Stem vs Root) yielded Q² values ranging from 0.992 to 0.995, R²X from 0.804 to 0.860, and R²Y of 1 for all comparisons, indicating excellent predictive performance and reliable group separation (see Supplementary Table 3 in S1 File). DAMs were defined by VIP > 1, |log_2_FC| ≥ 1, and FDR < 0.05, from which DSMs were selected. KEGG annotation was then applied to the DSMs. To investigate the dynamic accumulation patterns of DSMs, fuzzy c-means clustering was performed using the Mfuzz package in R. Metabolites were clustered based on their relative abundance profiles, with the optimal cluster number determined by minimizing the cluster centroid distance [27].

### 2.5. RNA extraction, library construction, and sequencing

RNA isolation was carried out using the TianGen Total RNA Kit (Beijing, China). Briefly, liquid nitrogen-ground tissues were homogenized, and total RNA was eluted in 50 µL of DEPC-treated water. RNA concentration and integrity were measured with a Qubit fluorometer and a Qsep400 bioanalyzer (Thermo Fisher Scientific, USA; Phonton Biotech, Taiwan, China), respectively. For library preparation, poly(A)-containing mRNA was captured using oligo(dT)-coated magnetic beads and reverse-transcribed into cDNA with the PrimeScript™ RT Kit (TaKaRa, China). Sequencing adapters were ligated to both ends of the cDNA, and the resulting libraries were barcoded for multiplexing. The libraries were constructed as non-stranded (unstranded) using the standard double-stranded cDNA synthesis protocol with random hexamer priming. After adapter ligation, library concentration and size distribution were re-assessed using the Qubit and Qsep400 systems. Qualified libraries were then subjected to cluster generation on a cBot instrument with the TruSeq PE Cluster Kit v3 (Illumina), followed by paired-end sequencing (125 or 150 bp) on an Illumina HiSeq platform [28]. Raw sequence data are accessible at NCBI SRA under accession number PRJNA1242508.

### 2.6. Data analysis of RNA-seq

Raw reads were processed with fastp (v0.23.2) to remove low-quality bases, adapters, and ambiguous reads [29]. Clean reads from all samples were then assembled together using Trinity (v2.15.1) in a merged assembly mode, which improves detection of both highly and weakly expressed transcripts and yields a more complete transcriptome reference. After assembly, Corset (v1.09) was used to cluster and remove redundant transcripts. Functional annotation of the non-redundant transcripts was performed by aligning them against KEGG, NR, Swiss-Prot, GO, KOG, and TrEMBL databases using DIAMOND (v2.1.6). Transcript abundance was estimated with RSEM (v1.3.3), producing FPKM values normalized by transcript length and total mapped reads. Differential expression analysis between tissue pairs was conducted with DESeq2 (v1.47.1), applying thresholds of |log_2_FC| ≥ 1 and FDR < 0.05 to identify DEGs [30]. Putative transcription factors among DEGs were predicted using iTAK (v2.0.2). Additionally, Mfuzz clustering was applied to DEGs following the same procedure used for DSMs [31].

### 2.7. Integrated analysis of transcriptomic and metabolomic data

Based on a comparison of the Mfuzz analysis results of DEGs and DSMs, we discovered DEGs clusters exhibiting expression trends across root, stem, leaf, and flower of *Eupatorium lindleyanum* that were consistent with the accumulation patterns of the corresponding metabolites. These selected genes were subsequently integrated into a weighted gene co-expression network analysis (WGCNA). The gene co-expression network was constructed in R using the WGCNA package. Co-expression modules were generated with the blockwise Modules function, adhering to default parameters except for three modifications: the soft-thresholding power was set to 18, the mergeCutHeight to 0.25, and the minModuleSize to 50. Following normalization of the FPKM values, an adjacency matrix was created and subsequently converted into a topological overlap matrix (TOM) [32]. Co-expression modules were then identified by grouping genes with similar expression patterns into discrete clusters. We then identified the characteristic genes within these modules, focusing on their expression level and their associations with the synthesis of sesquiterpenoid metabolites. Following WGCNA, ten sesquiterpenoid metabolites (Isoalantolactone, Shizukanolide A, Britannilactone, Eupalinolide B, Dehydrosaussurea lactone, Yomogin, Shizukanolide H, Magnograndiolide, Costunolide, and Dihydrocostunolide) were selected for subsequent analysis based on their significant differential accumulation across *Eupatorium lindleyanum* tissues and their well-documented bioactivities [33–41]. Pearson correlation analysis was performed between the abundance of each metabolite and the module eigengene values from WGCNA. Modules exhibiting an absolute correlation coefficient > 0.8 with a statistical significance of *P* < 0.05 were retained for further investigation. Genes within the selected modules were then subjected to KEGG enrichment and cluster analysis. Finally, to pinpoint key regulatory candidates, genes annotated under the terpenoid backbone biosynthesis (ko00900) and sesquiterpenoid biosynthesis (ko00909) pathways were specifically retained and identified [42].

### 2.8. Quantitative real-time PCR validation of candidate genes

The specific primers for the 12 candidate genes and a reference gene were designed (Primer Premier 5.0), and their amplification efficiencies were validated (see Supplementary Table 1 in S1 File). Quantitative real-time PCR (qRT-PCR) was performed using the QuantiNova SYBR Green PCR Kit (QIAGEN, Hilden, Germany) on an ABI 7500 real-time PCR system (Thermo Fisher, USA). The 10 μL reaction mixture contained 5 μL of 2 × SYBR Green PCR Master Mix, 0.05 μL of QN ROX Reference Dye, 0.7 μL each of forward and reverse primers (0.7 μM final concentration), 1 μL of cDNA template (≤100 ng), and 2.55 μL of RNase-free water. The thermal cycling protocol was as follows: initial denaturation at 95°C for 2 min, followed by 40 cycles of denaturation at 95°C for 5 s and combined annealing/extension at 60°C for 30 s. A melting curve analysis was performed after amplification to verify product specificity. The β-actin gene served as the internal reference, and relative expression levels were calculated using the 2^−ΔΔCt^ method. Each stage of the experiment involved three biological replicates and three technical replicates. The β-actin gene was subjected to stability assessment using three software tools: geNorm version 3.5, NormFinder version 0.953, and BestKeeper version 1.0. The M value calculated by geNorm was 0.527, the stability value determined by NormFinder was 0.082, and the standard deviation value computed by BestKeeper was 0.453. The analysis results indicate that β-actin exhibits good expression stability and can be used as a reference gene.

### 2.9. Statistical analysis and data visualization

The metabolomic and transcriptomic datasets were initially stored and processed using Microsoft Excel 2021. Correlation analyses were conducted with SPSS software (version 26.0, IBM, USA). Principal component analysis, hierarchical clustering, and Mfuzz temporal expression analysis were performed, and corresponding visualizations were generated, utilizing the corrplot and base packages in R (version 4.2.1). Bar charts and additional figures were prepared with OriginPro software (version 2022b, OriginLab Corporation, USA).

## 3. Result

### 3.1. Profiling of sesquiterpenoid metabolites in *Eupatorium lindleyanum* derived from four different tissues

Using UPLC-MS/MS, a total of 321 sesquiterpenoid metabolites were identified in *Eupatorium lindleyanum* (Supplementary Table 2 in S1 File). The comparative total relative content of these metabolites in four distinct tissues is presented in Fig 1A. Sesquiterpenoid metabolites predominantly accumulated in the flower of *Eupatorium lindleyanum*, followed by the leaf. In contrast, their contents in the root and stem were significantly lower. This pattern indicates a gradual increase in sesquiterpenoid accumulation from root and stem to leaf and flower. Principal component analysis (PCA) of twelve samples (including three replicates) revealed that samples from different tissue parts were distinctly separated into independent principal components (PC1, 50.58%; PC2, 31.17%), indicating significant differences in sesquiterpenoid metabolites among various tissues of *Eupatorium lindleyanum* (Fig 1B). The correlation heatmap demonstrated strong intra-group consistency, with biological replicates of each tissue type exhibiting high correlation coefficients (typically >0.9). In contrast, inter-group comparisons revealed that root exhibited relatively weak correlations with stem, leaf, and flower, whereas correlations among the above-ground tissues (stem, leaf, and flower) were relatively strong. This suggests a clear metabolic distinction between root and above-ground tissues, while the metabolic profiles of stem, leaf, and flower are more similar to one another. (Fig 1C). To assess metabolite-specific variation, hierarchical clustering analysis (HCA) was performed using the relative abundances of sesquiterpenoids across distinct plant tissues (Fig 1D). Hierarchical clustering analysis further substantiated the above findings, revealing distinct separation among tissue types. Root, stem, leaf, and flower each contained differentially abundant sesquiterpenoid metabolites. In summary, the profiling of sesquiterpenoid metabolites revealed a clear tissue-specific pattern in *Eupatorium lindleyanum*, providing a basis for subsequent differential metabolites analysis.

**Fig 1:**
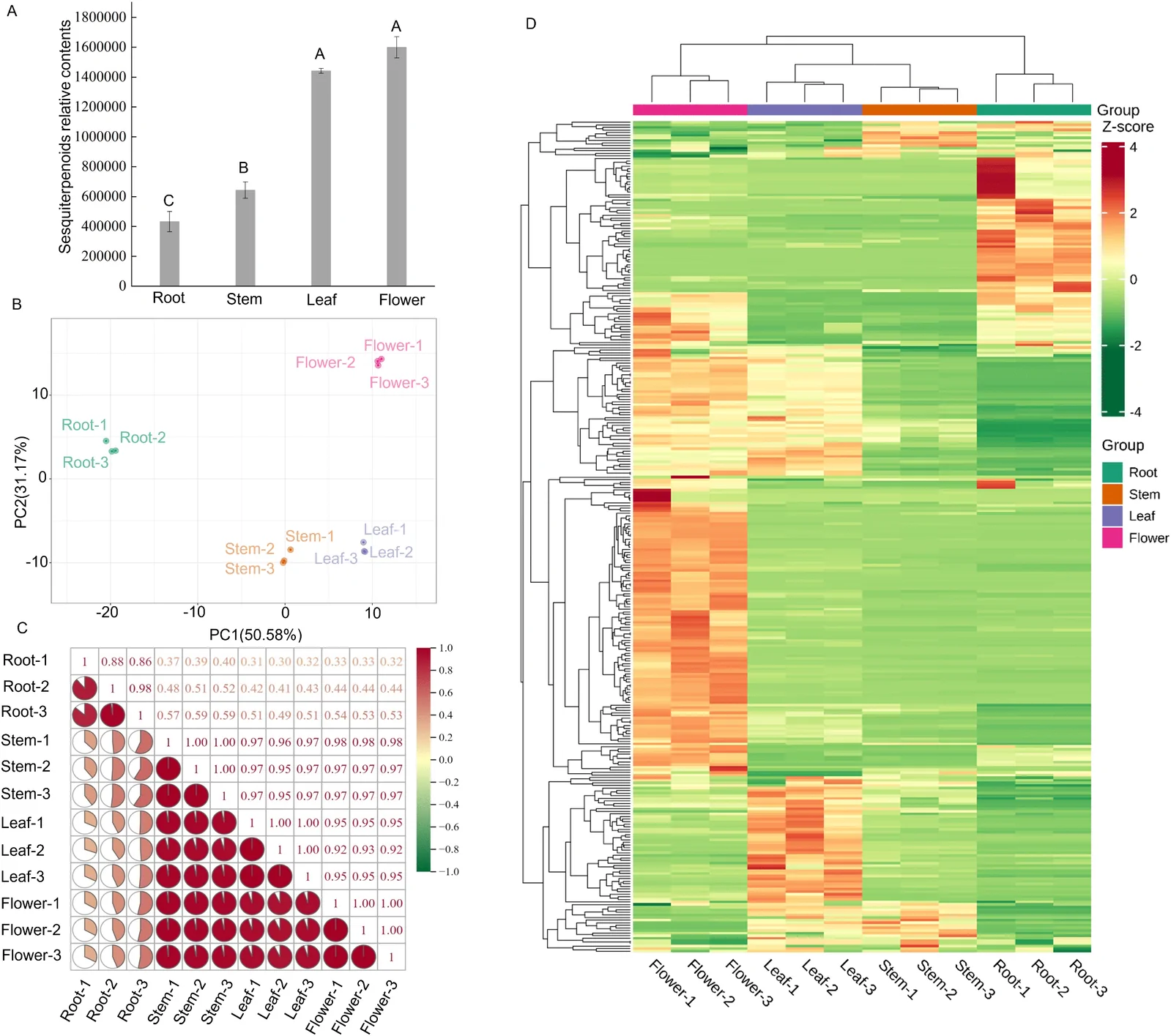
Analysis of sesquiterpenoid metabolites in different tissues of *Eupatorium lindleyanum.* (A) Total sesquiterpenoid content from root, stem, leaf and flower, different letters denote statistically significant differences among tissues (*P* < 0.05). (B)Principal component analysis of different tissues from *Eupatorium lindleyanum*. (C) Pearson correlation analysis between 12 samples. (D) Hierarchical clustering analysis of 321 sesquiterpenoid metabolites in 12 samples: color intensity reflects relative abundance.

### 3.2. Differentially accumulated sesquiterpenoid metabolites in four tissues of *Eupatorium lindleyanum*

299 DSMs were identified by screening DAMs across all pairwise tissue comparisons: flower vs. root, flower vs. stem, flower vs. leaf, stem vs. root, leaf vs. root, and leaf vs. stem (Supplementary Table 3 in S1 File). Among the six tissues pairwise comparisons in *Eupatorium lindleyanum*, the flower vs. root comparison yielded the highest number of DSMs, with 185 upregulated and 48 downregulated metabolites. A similar pattern was observed in the leaf vs. root comparison, which showed 146 upregulated and 76 downregulated metabolites. The stem vs. root and flower vs. stem comparisons exhibited comparable DSM counts, with 122 upregulated and 88 downregulated and 185 upregulated and 23 downregulated, respectively. In the flower vs. leaf comparison, 146 metabolites were upregulated and 51 were downregulated. The leaf vs. stem comparison contained the fewest DSMs, with only 124 upregulated and 16 downregulated metabolites. Among these differentially accumulated metabolites, 28 DSMs overlapped across all six pairwise comparisons (Fig 2A). These 28 common DSMs represent a conserved metabolic core, likely involved in essential functions across tissues (Supplementary Table 4 in S1 File). Crucially, the vast majority of DSMs were tissue-specific, revealing profound metabolic divergence between the root and aerial organs. This high degree of specialization reflects distinct physiological roles governed by organ-specific gene expression during development. Mfuzz analysis classified the 299 DSMs into three clusters, each exhibiting a distinct tissue-specific accumulation pattern in *Eupatorium lindleyanum*. In sub cluster 1, DSMs demonstrated the highest relative abundance in leaf compared to other tissues. In sub cluster 2, the highest abundance was observed in flower, while sub cluster 3 was characterized by a predominant accumulation of DSMs in root (Fig 2B). The clustering results of metabolites within each sub-cluster consistently reinforce this tissue-specific partitioning, corroborating the organ-specific metabolic profiles observed in the overall analysis.

**Fig 2:**
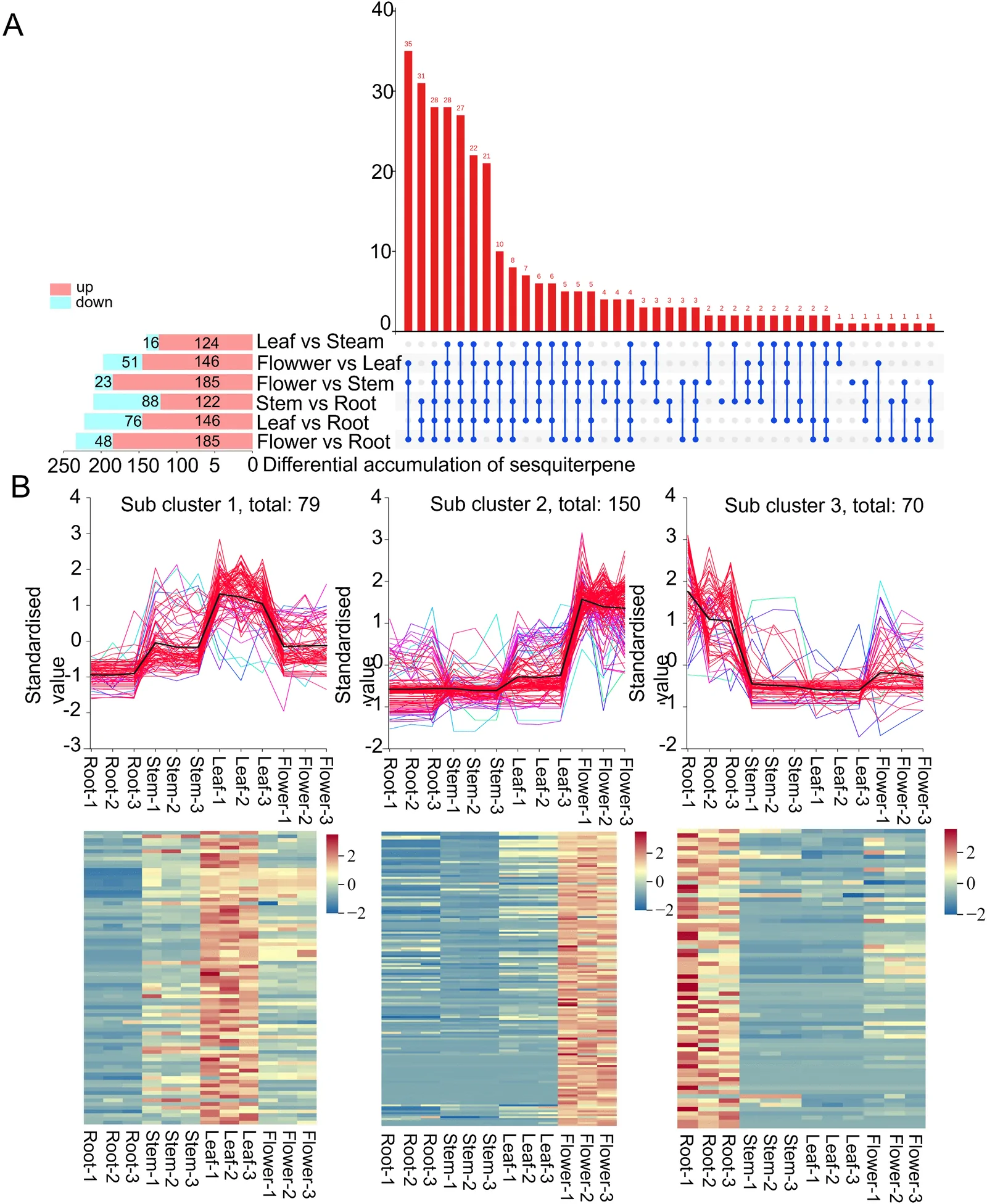
Differential analysis of sesquiterpenoids across tissues in *Eupatorium lindleyanum.* (A) Visualization of differentially accumulated sesquiterpenoid metabolites: a bar chart (left) and a corresponding node graph (right). (B) Cluster analysis of sesquiterpenoid accumulation patterns. (Top) Fuzzy c-means clustering (Mfuzz) identified three distinct metabolite clusters. The cluster centroid is shown as a black line, with remaining lines representing individual compounds. (Bottom) Accumulation levels of sesquiterpenoids belonging to each cluster.

### 3.3. Transcriptome profiling analysis of *Eupatorium lindleyanum* in four distinct tissues

A total of 12 cDNA libraries were prepared from four tissues (root, stem, leaf, flower) of *Eupatorium lindleyanum*, each with three biological replicates. Sequencing generated 47983328–69716676 raw reads per library, and after quality filtering, 47182646–66154460 clean reads were obtained per sample, totaling 99.43 Gb of clean data. The Q20 and Q30 values across all samples were ≥98.38% and ≥95.35%, respectively, with GC content ranging from 42.79% to 44.1%(Supplementary Table 5 in S1 File). De novo assembly produced 147743 unigenes, of which 66.54% were annotated in at least one public database (NR: 90758; TrEMBL: 91058; GO: 80518; KEGG: 70028; SwissProt: 65385; KOG: 60040) (Supplementary Table 6 in S1 File). PCA of FPKM-normalized expression values showed that PC1 and PC2 explained 31.60% and 23.12% of the variance, respectively, with clear separation among tissue groups and high intra-group consistency (Fig 3A). Correlation analysis further confirmed strong repeatability within replicates, and a notable correlation was observed between stem and leaf transcript profiles (Fig 3B). The high-quality transcriptome profile, characterized by clear tissue segregation and strong reproducibility, provides a robust foundation for subsequent differential and integrated analyses.

**Fig 3:**
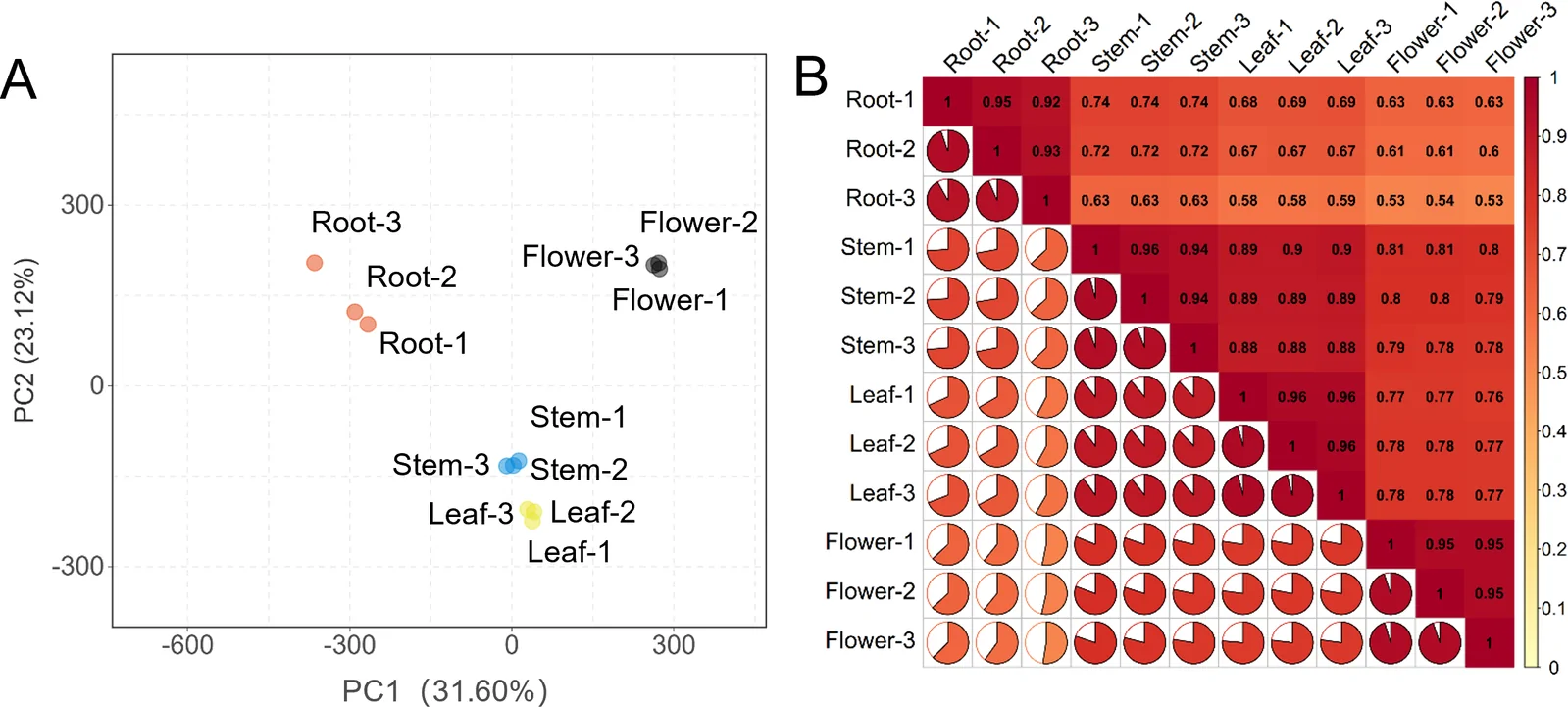
Principal component and correlation analyses of the *Eupatorium lindleyanum* transcriptome across four tissues. (A) PCA score plot. (B) Correlation heatmap of samples. All analyses included biological replicates (n = 3) for root, stem, leaf, and flower tissues.

### 3.4. Analysis of differentially expressed genes

Based on the threshold of |log2FC| ≥ 1 and FDR < 0.05, a total of 53610 differentially expressed genes (DEGs) were identified through pairwise comparisons across the six tissue combinations, with detailed information provided in Supplementary Table 7 in S1 File. Fig 4A shows that 519 DEGs were common among all six pairs of comparisons. Fig 4B displays the quantities of differentially expressed genes (DEGs) detected in pairwise tissue comparisons. These analyses identified 24654 DEGs between flower and leaf tissues (14761 upregulated, 9893 downregulated), 37090 between flower and root (17943 upregulated, 19147 downregulated), 24617 between stem and leaf (17992 upregulated, 6625 downregulated), 26984 between stem and root (11021 upregulated, 15963 downregulated), 32461 between leaf and root (13661 upregulated, 18800 downregulated), and 21331 between flower and stem (12448 upregulated, 8883 downregulated). To explore potential genes involved in secondary metabolism, DEGs were analyzed via GO enrichment. Terms related to the mevalonate pathway and ribosome/cofactor activities were enriched. This suggests that DEGs may support the cellular infrastructure for sesquiterpenoid biosynthesis, though direct roles require further validation (Fig 4C). Based on expression levels, the 53610 DEGs were classified into six clusters (Fig 4D). Genes in Cluster 1 showed relatively high expression in stem, those in Cluster 3 exhibited a gradually increasing expression trend across root, stem, leaf, and flower, and those in Cluster 6 displayed relatively high expression in root. Notably, the expression patterns of Clusters 2, 4, and 5 resembled those of DSM clusters 1, 2, and 3. Specifically, DEGs in Cluster 2 exhibited the highest expression in leaf, Cluster 4 in flower, and Cluster 5 showed a gradual decrease in expression from root to flower. This parallel expression pattern suggests that DEGs sharing similar tissue-specific trends with DSMs are likely key candidates involved in sesquiterpenoid biosynthesis and its regulation. Subsequently, KEGG functional enrichment analysis was performed on DEGs from Clusters 2, 4, and 5. Significantly, DEGs within each cluster were enriched in pathways related to sesquiterpenoid biosynthesis and terpenoid backbone biosynthesis(Fig 4E).

**Fig 4:**
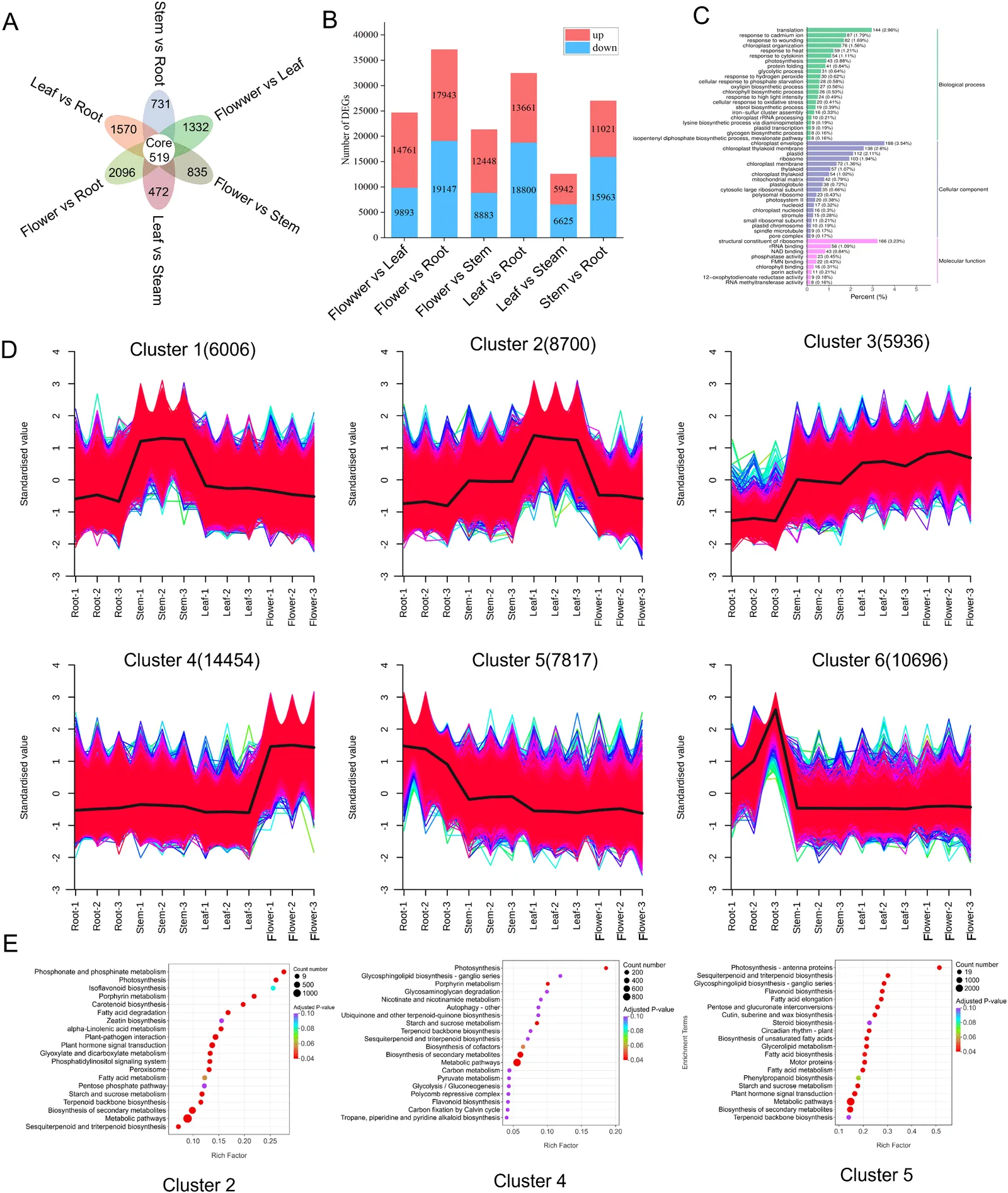
Analysis of differentially expressed genes (DEGs) in *Eupatorium lindleyanum* across different tissues. (A) Venn diagram of DEGs in the six pairs of comparisons. (B) Number of DEGs in the six pairs of comparisons. (C) GO enrichment analysis of DEGs. (D) Cluster analysis of differentially expressed genes (DEGs). All DEGs were categorized into six distinct clusters based on their expression patterns using the Mfuzz R package. (E) KEGG enrichment analysis of DEGs in *Eupatorium lindleyanum* from Clusters 2、Clusters 4 and Clusters 5.

### 3.5. Identification of sesquiterpenoid biosynthesis-related genes through WGCNA-based co-expression network analysis

To further explore genes related to sesquiterpenoid biosynthesis, 30971 DEGs exhibiting expression patterns consistent with DSMs across different tissues were consolidated and subjected to WGCNA. These genes were grouped into 13 distinct color-coded modules (with different colors representing different modules; the gray module contains the remaining unrelated genes) (Fig 5A). Module–metabolites correlation analysis showed that seven modules—black, green, magenta, yellow, pink, red, and turquoise—were significantly correlated with the 10 DSMs (correlation coefficient > 0.8). Accordingly, these seven modules were selected for further analysis (Fig 5B). The expression levels of genes contained in each module are presented in Supplementary Figure 2 in S1 File. Genes highly expressed in flower were predominantly clustered in the black, green, magenta, and turquoise modules. Those with high expression in leaf were mainly enriched in the pink and yellow modules. Genes showing relatively high expression in stem, leaf, and flower were primarily assigned to the red module. Furthermore, the genes included in the seven selected modules were subjected to KEGG enrichment analysis respectively (Fig 5C). All modules except the pink one were annotated to the KEGG pathways: metabolic pathways(ko01100), biosynthesis of secondary metabolitess(ko01110), sesquiterpenoid and triterpenoid biosynthesiss(ko00909), and terpenoid backbone biosynthesiss(ko00900). Moreover, based on the WGCNA results, Correlation network diagrams were constructed for the genes within each module. Genes strongly correlated with the hub genes (edge weight ≥ 0.3) are shown in Supplementary Figure 3 in S1 File. Within the black, green, magenta, yellow, pink, red, and turquoise modules, a total of 8, 8, 8, 5, 8, 8, and 5 genes, respectively, were identified as hub genes with high topological importance and exhibited strong correlations with other genes in the network. These results indicate that the hub genes are key players involved in the biosynthesis of sesquiterpenoid metabolites in *Eupatorium lindleyanum*. Within the seven modules, genes mapped to ko00909 and ko00900 were selected and merged, yielding a total of 20 genes (88 unigenes) associated with sesquiterpenoid and terpenoid backbone biosynthesis, namely, *GAO*, *GAS*, *PMK*, *ACAT*, *HMGCS*, *HMGCR*, *MVK*, *MVD*, *FDPS*, *IDI*, *dxs*, *dxr*, *ispD*, *ispE*, *ispF*, *ispG*, *ispH*, *FLDH*, *NES1*, *GDS*, (Supplementary Table 8 in S1 File). These genes have been proposed as key players in regulating sesquiterpenoid biosynthesis in *Eupatorium lindleyanum*; however, their functional characterization still requires further investigation.

**Fig 5:**
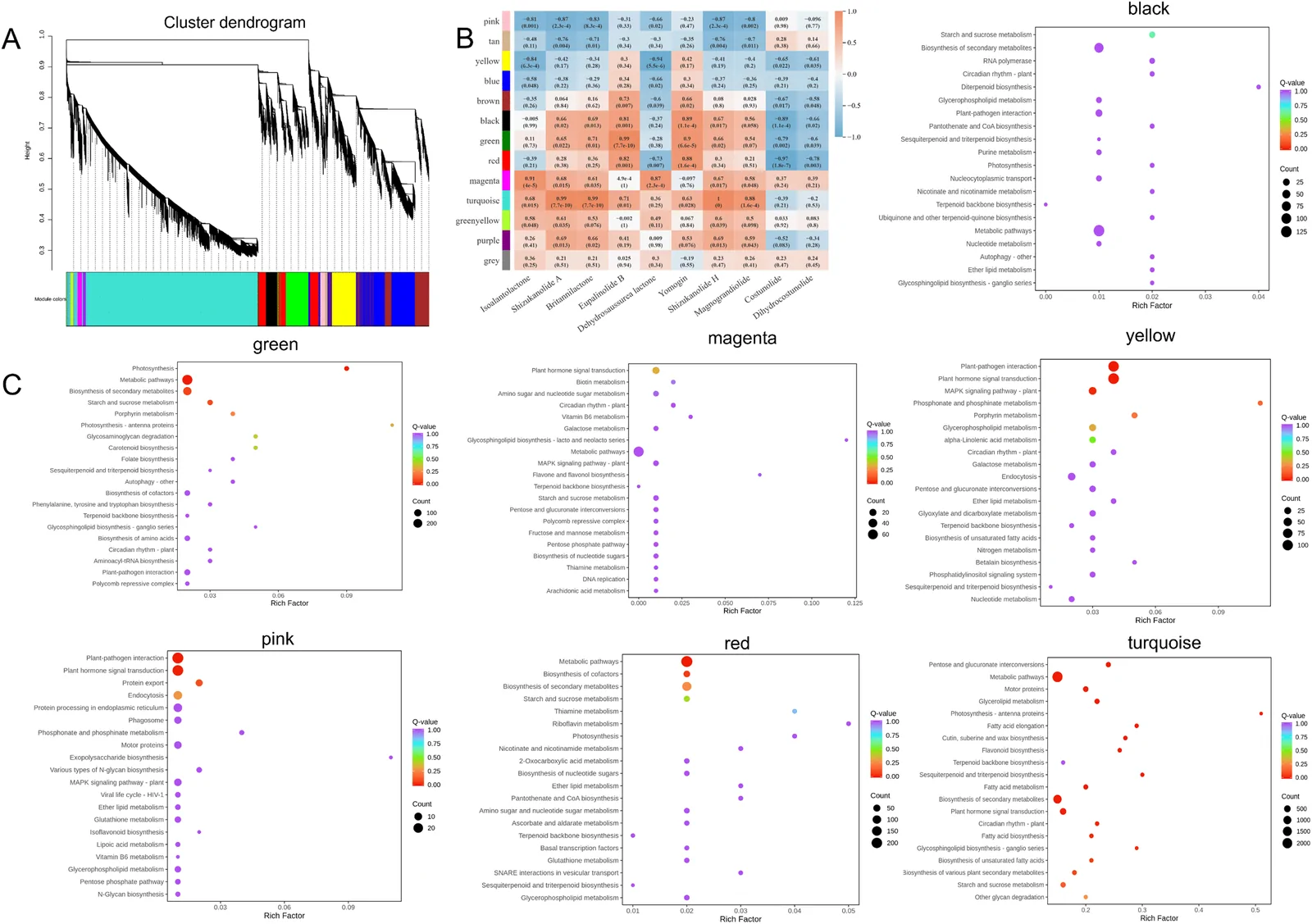
Weighted gene co-expression network analysis (WGCNA) of DEGs in Clusters 2, 4, and 5. (A) Cluster dendrogram of co-expressed genes. Each leaf represents a single gene, and the main branches correspond to 13 modules labeled with different colors. (B) Correlation heatmap between 13 module traits and 10 DSMs. Each cell at the intersection of a row and column represents the correlation coefficient. It is displayed based on the color scale on he right. The *P* values are displayed in the parentheses of each cell. (C) KEGG enrichment analysis of the genes in each module shows only the top 20 pathways with the most significant enrichment.

### 3.6. Construction of the sesquiterpenoid biosynthesis regulatory network in *Eupatorium lindleyanum*

Based on the results of WGCNA and KEGG enrichment analyses, the predicted sesquiterpenoid biosynthetic pathway is illustrated in Fig 6. The diagram illustrates the proposed biosynthetic route for sesquiterpenoids in *Eupatorium lindleyanum*, integrating both the mevalonate (MVA) and the 2-C-methyl-D-erythritol 4-phosphate (MEP) pathways, along with the subsequent enzymatic steps leading to the formation of sesquiterpenoid skeletons and their derivatives. In the MVA pathway, the sequential conversion of acetyl-CoA to isopentenyl diphosphate (IPP) is mediated by acetoacetyl-CoA thiolase (ACAT), hydroxymethylglutaryl-CoA synthase (HMGCS), hydroxymethylglutaryl-CoA reductase (HMGCR), mevalonate kinase (MVK), phosphomevalonate kinase (PMK), and diphosphomevalonate decarboxylase (MVD). Isopentenyl-diphosphate Delta-isomerase (IDI) subsequently catalyzes the reversible conversion of IPP to dimethylallyl diphosphate (DMAPP). In the MEP pathway, the initial steps involving pyruvate and glyceraldehyde-3-phosphate are catalyzed by 1-deoxy-D-xylulose-5-phosphate synthase (dxs) and 1-deoxy-D-xylulose-5-phosphate reductoisomerase (dxr), followed by a series of enzymatic reactions mediated by ispD, ispE, ispF, ispG, and ispH to ultimately generate IPP and DMAPP. The condensation of IPP and DMAPP into farnesyl diphosphate (FPP), the universal precursor of sesquiterpenoid, is catalyzed by farnesyl diphosphate synthase (FDPS). Following the formation of FPP, a diverse array of sesquiterpene synthases drive the structural diversification of terpenoid skeletons. These include germacrene A synthase (GAS), germacrene D synthase (GDS), and (3S,6E)-nerolidol synthase (NES1). Further modifications, such as oxidation and hydroxylation, are catalyzed by cytochrome P450s and dehydrogenases, including germacrene A oxidase (GAO), and NAD + -dependent farnesol dehydrogenase (FLDH). Taken together, Fig 6 presents a data-driven reconstruction of the sesquiterpenoid biosynthetic pathway in *Eupatorium lindleyanum*, in which each enzymatic step is supported by WGCNA-prioritized unigenes belonging to trait-associated modules. This approach not only consolidates fragmented transcriptome annotations into a coherent metabolic map, but also establishes a prioritized candidate list for subsequent functional characterization and genetic manipulation.

**Fig 6:**
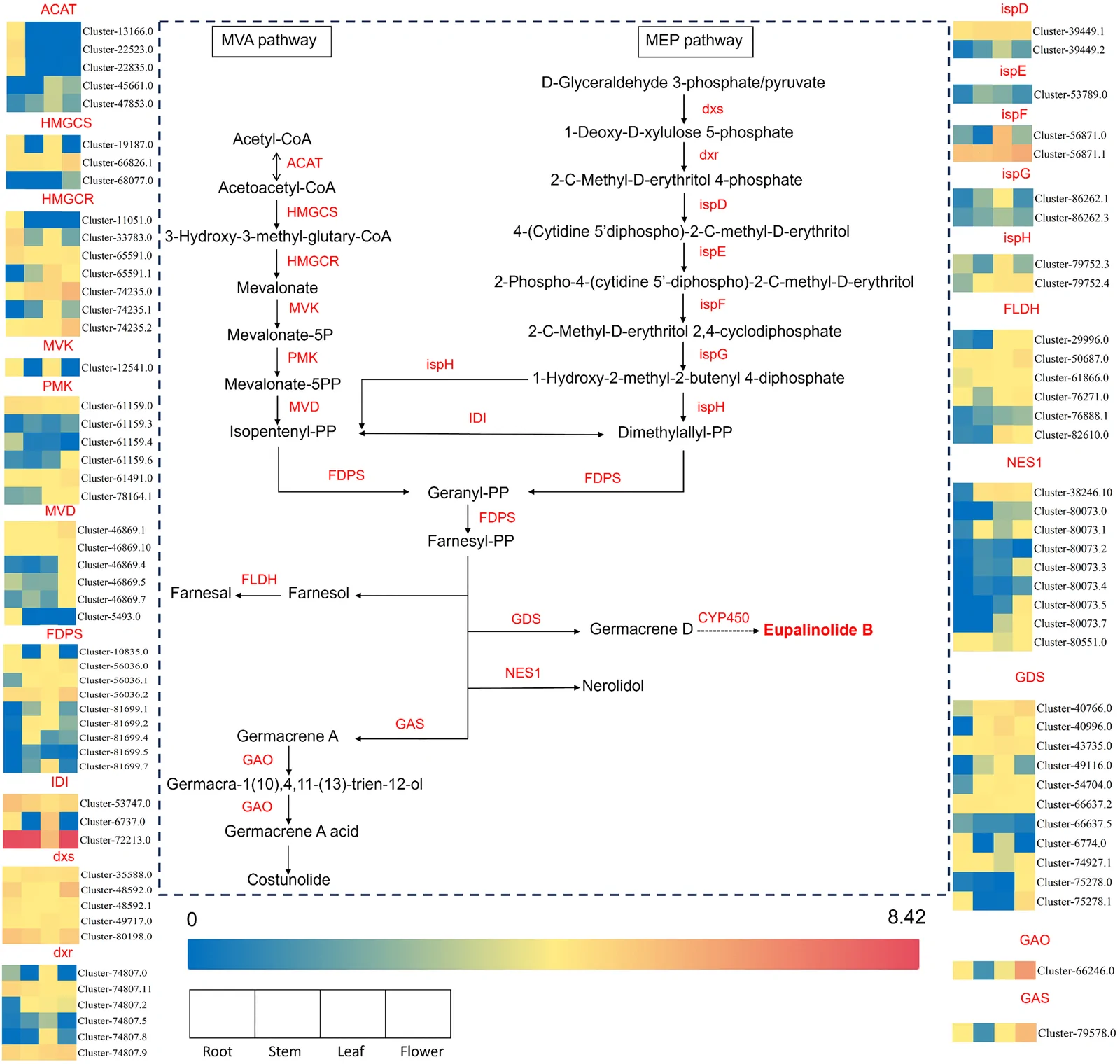
Proposed pathway for sesquiterpenoid biosynthesis in *Eupatorium lindleyanum.* The red, blue and yellow rectangles indicate levels of gene expression. MVA: mevalonate pathway; MEP: 2-C-methyl-D-erythritol 4-phosphate pathway; GAO: germacrene A oxidase; GAS: germacrene A synthase; PMK: phosphomevalonate kinase; ACAT: acetyl-CoA acetyltransferase; HMGCS: hydroxymethylglutaryl-CoA synthase; HMGCR: hydroxymethylglutaryl-CoA reductase; MVK: mevalonate kinase; MVD: diphosphomevalonate decarboxylase; FDPS: farnesyl diphosphate synthase; IDI: isopentenyl-diphosphate Delta-isomerase; dxs: 1-deoxy-D-xylulose-5-phosphate synthase; dxr: 1-deoxy-D-xylulose-5-phosphate reductoisomerase; ispD: 2-C-methyl-D-erythritol 4-phosphate cytidylyltransferase; ispE: 4-(cytidine 5’-diphospho)-2-C-methyl-D-erythritol kinase; ispF: 2-C-methyl-D-erythritol 2,4-cyclodiphosphate synthase; ispG: (E)-4-hydroxy-3-methylbut-2-enyl-diphosphate synthase; ispH: 4-hydroxy-3-methylbut-2-en-1-yl diphosphate reductase; FLDH: NAD + -dependent farnesol dehydrogenase; NES1: (3S,6E)-nerolidol synthase; GDS: germacrene D synthase.

### 3.7. Analysis of transcription factors associated with sesquiterpenoid biosynthesis

To investigate the regulatory mechanisms underlying tissue-specific accumulation of sesquiterpenoid metabolites in *Eupatorium lindleyanum*, we characterized transcription factors exhibiting differential expression (|log_2_ fold change| ≥ 1, FDR < 0.05) across four tissues and assessed their association with the abundance of ten distinct sesquiterpenoids. Significantly correlated transcription factor–metabolite pairs were identified based on stringent thresholds (|R| > 0.8, *P* < 0.05) (Fig 7). Among the five transcription factor families regulating sesquiterpenoid biosynthesis, all exhibited substantial associations with sesquiterpenoids, with cluster numbers ranging from 15 (*NAC*) to 23 (*MYB*). Specifically, the *MYB* family showed the highest number of significantly correlated clusters (23 clusters with 8 metabolites), followed by *bHLH* (21 clusters with 9 metabolites), *AP2/ERF* (20 clusters with 10 metabolites), *WRKY* (19 clusters with 9 metabolites), and *NAC* (15 clusters with 8 metabolites). The differences in the expression patterns of the transcription factors among the four tissues are presented in Suplementary Figure 4 in S1 File. These results suggest that these transcription factors play important roles in regulating sesquiterpenoid biosynthesis in different tissues of *Eupatorium lindleyanum*. While these correlation-based findings provide a robust framework for prioritizing candidate regulators, experimental validation—such as gene silencing or overexpression combined with metabolite profiling—is warranted to establish causal relationships between specific transcription factors and sesquiterpenoid biosynthesis.

**Fig 7:**
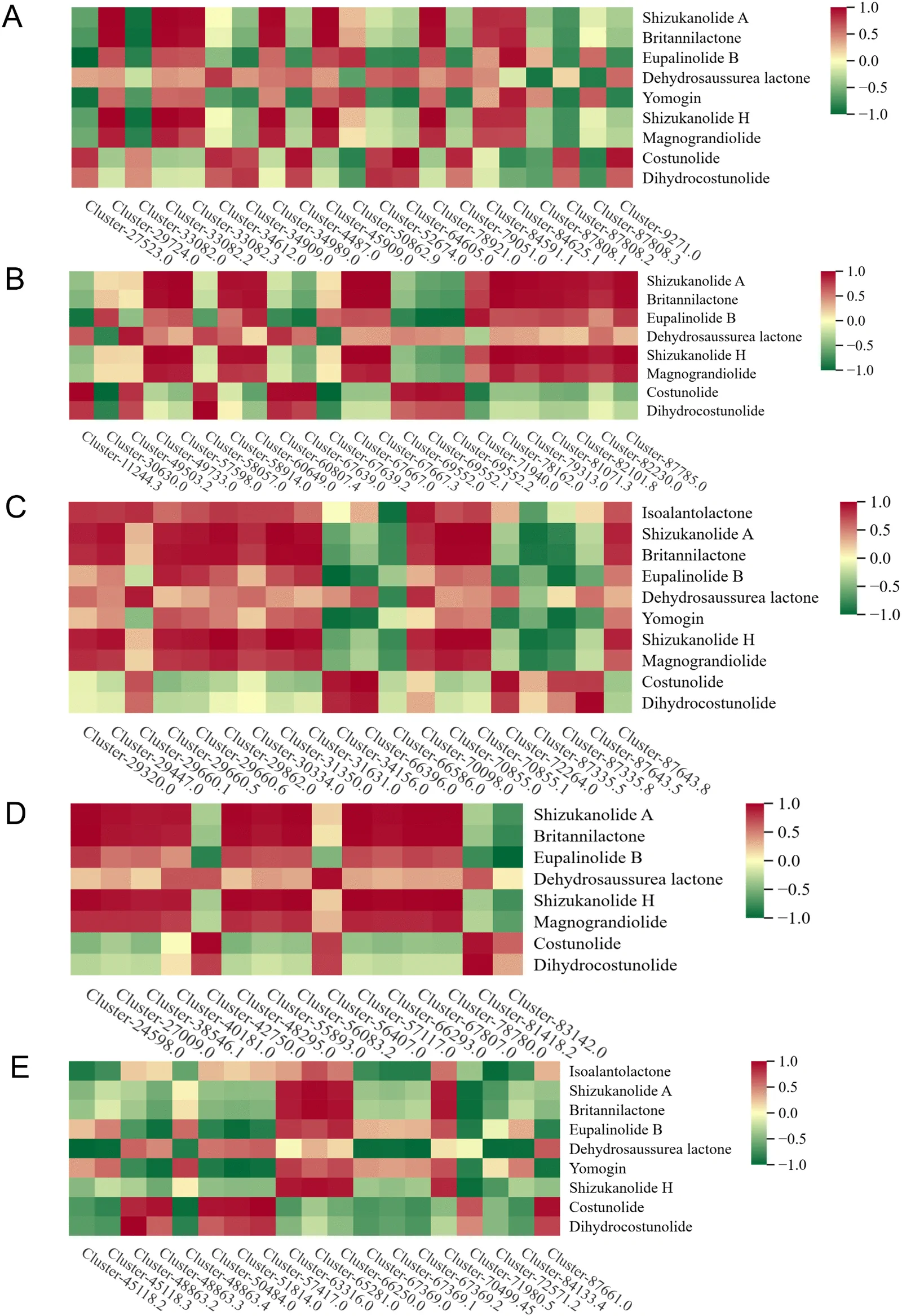
Correlation analysis between transcription factors and DSMs in *Eupatorium lindleyanum* (|R| > 0.8, *P* < 0.05); A: *bHLH*, B: *MYB,* C: *AP2/ERF*, D: *NAC*; E: *WRKY.* The color intensity of each grid cell reflects the strength of the correlation coefficient.

### 3.8. qRT‒PCR validation of candidate genes in the sesquiterpenoid biosynthesis pathway

To assess the reliability of the transcriptome data, the expression patterns of 12 candidate genes were examined using qRT-PCR. Their relative expression across the four examined tissues of *Eupatorium lindleyanum* showed a consistent trend with the FPKM values obtained from RNA-Seq. (Fig 8). Pearson correlation analysis revealed that eight of these genes exhibited significant positive correlations between the qRT-PCR and RNA-Seq datasets: *GAS* (r = 0.998, *P* = 0.002), *GAO* (r = 0.996, *P* = 0.004), *MVD* (r = 0.997, *P* = 0.003), *dxs* (r = 0.991, *P* = 0.009), *ACAT* (r = 0.984, *P* = 0.016), *HMGCS* (r = 0.978, *P* = 0.022), *MVK* (r = 0.967, *P* = 0.033), and *PMK* (r = 0.963, *P* = 0.037). By contrast, *IDI* exhibited a significant negative correlation (r = –0.963, *P* = 0.037), while *HMGCR*, *FDPS*, and dxr did not show statistically significant correlations (*P* > 0.05). This high concordance for the majority of genes (66.67%) validates the robustness of the RNA-Seq dataset and confirms its suitability for downstream expression analyses. The consistency between the two platforms further supports the fidelity of the transcriptomic profiling and the reliability of the bioinformatic inferences derived from it.

**Fig 8:**
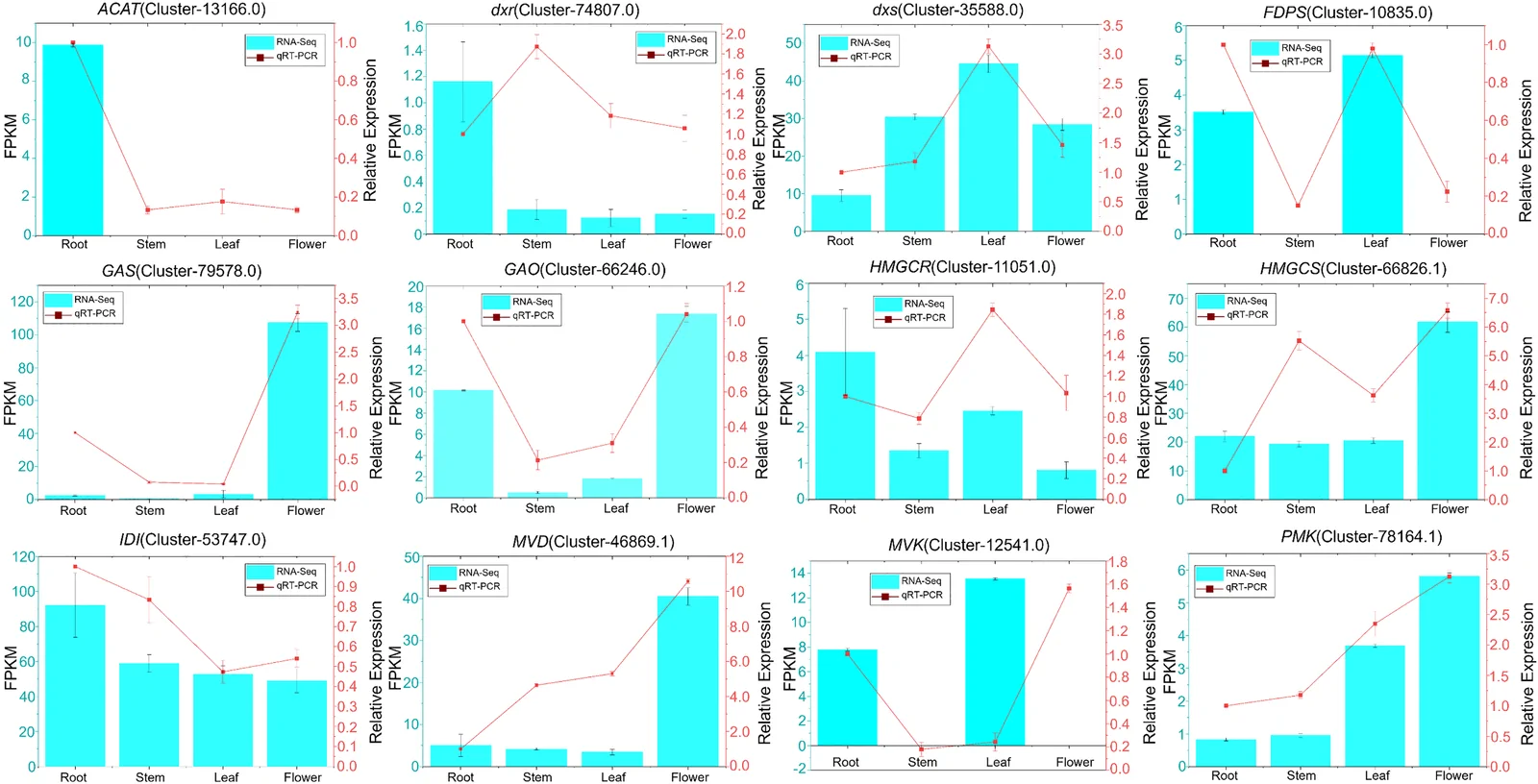
qRT‒PCR validation of 12 candidate genes. The horizontal axis represents the four tissues of *Eupatorium lindleyanum*, the right vertical axis represents the relative expression levels of genes determined via qRT‒PCR, and the left vertical axis represents the FPKM values of the genes.

## 4. Discussion

The medicinal value of *Eupatorium lindleyanum* derives largely from its repertoire of bioactive sesquiterpenoids. While these compounds have been extensively studied for their pharmacological properties, the spatial organization of their biosynthesis within the plant—and the genes that govern it—has received far less attention [43]. This study aimed to fill that gap by integrating tissue-specific metabolomic and transcriptomic profiles. We integrated UPLC–MS/MS-based metabolomics with RNA-Seq transcriptomics to comprehensively profile sesquiterpenoid metabolites and their associated gene expression signatures across root, stem, leaf, and flower tissues. Through weighted gene co-expression network analysis (WGCNA) coupled with Mfuzz trend clustering, we identified structural genes and transcription factors highly correlated with the accumulation of key sesquiterpenoids and constructed a regulatory network model for sesquiterpenoid biosynthesis in this medicinal plant.

### 4.1. Tissue-specific accumulation of sesquiterpenoids and its transcriptional basis

In this study, we identified 321 sesquiterpenoid metabolites in *Eupatorium lindleyanum*, including various sesquiterpene lactones, sesquiterpene hydrocarbons, and their oxygenated derivatives. Notably, compounds such as eupalinolide B, zaluzanin C, isoalantolactone, dehydrocostus lactone, costunolide, spathulenol, and atractylenolide I/II have been previously characterized in other Asteraceae species, confirming the consistency of our findings with earlier reports [44]. Principal component analysis (PCA) and hierarchical clustering analysis revealed distinct tissue-specific signatures of these metabolites in root, stem, leaf, and flower. In root and stem, the relative abundance of sesquiterpenoids was generally low, with only a few metabolites showing high abundance. For example, Agalleudesmanol D (Flower vs Root Log_2_FC = −7.98; Leaf vs Root Log_2_FC = −7.96; Stem vs Root Log_2_FC = −7.66), Calacorene oxide (Flower vs Root Log_2_FC = −7.22; Leaf vs Root Log_2_FC = −7.09; Stem vs Root Log_2_FC = −6.96), and Curcumafuranol (Flower vs Root Log_2_FC = −3.27; Leaf vs Root Log_2_FC = −3.76; Stem vs Root Log_2_FC = −5.71) were highly abundant in root. Similarly, 13-Nor-desacylcynaropicrin (Flower vs Stem Log_2_FC = −3.45; Leaf vs Stem Log_2_FC = −0.29; Stem vs Root Log_2_FC=5.88) and Oxyphyllenone A (Flower vs Stem Log_2_FC = −2.59; Leaf vs Stem Log_2_FC = −4.59; Stem vs Root Log_2_FC=2.04) were elevated in stem. In contrast, the leaf and flower contained relatively high levels of most sesquiterpenoids, with particularly elevated concentrations observed in the flower. This pattern suggests that the accumulation of sesquiterpenoids is tissue-specific and may be regulated by specific genes, as further explored in our integrative metabolomic and transcriptomic analysis below [45]. The tissue-specific variation in sesquiterpenoid metabolic profiles reflects their distinct physiological functions and adaptation to differential environmental exposures [46]. Root is involved primarily in nutrient absorption and belowground defense, whereas leaf and flower are exposed to relatively high levels of environmental stressors, such as ultraviolet (UV) radiation, pathogen attack, and herbivory. The elevated accumulation of sesquiterpenoids in leaf and flower suggests their involvement in both ecological and physiological functions. These compounds may contribute to chemical defense against biotic and abiotic stresses, while certain volatile sesquiterpenoids could also act as attractants for pollinators. Among these, compounds such as costunolide, dehydrocostus lactone, isoalantolactone, eupalinolide B, and zaluzanin C have been previously documented as defense-related sesquiterpenoids that accumulate in response to biotic or abiotic elicitation in Asteraceae species. Meanwhile, volatile sesquiterpenoids such as spathulenol may serve dual functions in both direct defense and pollinator attraction [47,48]. Therefore, we speculate that the tissue-specific distribution of sesquiterpenoids in *Eupatorium lindleyanum* is regulated by specific genes [49]. Furthermore, we systematically compared the sesquiterpenoid profiles across four tissues of *Eupatorium lindleyanum* and identified 299 differentially accumulated sesquiterpenoid metabolites (DSMs). The highest number of DSMs was observed in the flower vs. root comparison, followed by leaf vs. root, indicating a pronounced metabolic divergence between belowground and aboveground organs. This suggests that the metabolic flux of sesquiterpenoids is differentially channeled in root compared to aerial tissues [50]. Notably, 28 DSMs were consistently differentially accumulated across all six pairwise comparisons. These common DSMs may represent a core set of sesquiterpenoids involved in essential physiological processes that are modulated across tissues. Further clustering analysis using Mfuzz categorized the DSMs into three major clusters with distinct tissue-preferred accumulation patterns. Cluster 1 comprised metabolites with highest abundance in leaf, suggesting a role in foliar defense against herbivores and environmental stress. Cluster 2 included metabolites predominantly accumulated in flower, which may function as volatile attractants for pollinators or as protectants for reproductive tissues. Cluster 3 was characterized by root-enriched metabolites, likely involved in belowground defense, allelopathy, or symbiosis. This tissue-specific partitioning of sesquiterpenoids aligns with the concept of metabolic specialization, wherein organs optimize their chemical profiles according to their unique physiological and ecological challenges [51]. The striking accumulation of DSMs in flower and leaf compared to root and stem further supports the hypothesis that aerial tissues are more metabolically active in sesquiterpenoid biosynthesis, possibly due to their direct exposure to biotic and abiotic pressures [52]. Importantly, the tissue-specific expression patterns of DEGs recapitulated those of DSMs. Specifically, DEGs from Clusters 2, 4, and 5 exhibited leaf-predominant, flower-predominant, and root-to-flower decreasing trends, respectively, mirroring the accumulation trajectories of DSM Clusters 1, 2, and 3. This concordant transcript–metabolite partitioning provided the rationale for selecting these three DEG clusters as the input for WGCNA, thereby enriching for genes most likely to be functionally associated with sesquiterpenoid biosynthesis.

### 4.2. Integration of transcriptomic and metabolomic data: structural genes underlying the biosynthetic pathway

In plants, the biosynthesis of sesquiterpenoids is primarily governed by structural genes within the corresponding metabolic pathway [53]. To uncover the molecular basis of this metabolic specialization, the integration of transcriptomic and metabolomic analyses revealed that a total of 20 structural genes encoding 88 unigenes were involved in the biosynthesis of sesquiterpenoids in *Eupatorium lindleyanum*. The genes were classified into three groups on the basis of their involvement in distinct biosynthetic pathway branches. Specifically, Group I genes are involved in the mevalonate (MVA) pathway, including *ACAT*, *HMGCS*, *HMGCR*, *MVK*, *PMK*, and *MVD*. The Group II genes are associated with the 2-C-methyl-D-erythritol 4-phosphate (MEP) pathway and include *dxs*, *dxr*, *ispD*, *ispE*, *ispF*, *ispG*, and *ispH*. Group III genes participate in the downstream biosynthesis and modification of sesquiterpenoids, including *FDPS*, *IDI*, *GAS*, *GDS*, *NES1*, *GAO*, and *FLDH*. In previous studies, most of these genes have been characterized and are well documented to play crucial roles in the biosynthesis of plant sesquiterpenoids. *ACAT*, *HMGCS*, and *HMGCR* are key enzyme genes in the MVA pathway that catalyze the conversion of acetyl-CoA to mevalonate, which provides the precursor for sesquiterpenoid biosynthesis [54]. *Dxs* and *dxr* participate in the MEP pathway, further contributing to the pool of isopentenyl diphosphate (IPP) and dimethylallyl diphosphate (DMAPP) [55,56]. *FDPS* is a core enzyme in the sesquiterpenoid biosynthesis pathway, catalyzing the condensation of IPP and DMAPP to form farnesyl diphosphate (FPP), the universal precursor for all sesquiterpenoids [57,58]. *GAS* and *GDS* are sesquiterpene synthases that generate germacrene A and germacrene D skeletons, respectively, providing key intermediates for downstream diversification [59]. NES1 catalyzes the formation of nerolidol, a volatile sesquiterpene alcohol with ecological functions [60]. *GAO* is cytochrome P450 monooxygenases that introduce oxygen-containing functional groups through oxidation and hydroxylation reactions, enhancing structural complexity and bioactivity [61]. *FLDH* catalyzes the oxidation of farnesol, further diversifying sesquiterpenoid profiles [62]. The coordinated action of these genes within the sesquiterpenoid biosynthesis pathway dictates the tissue-specific distribution of sesquiterpenoids in *Eupatorium lindleyanum*. Interestingly, the core structural genes appear to be functionally conserved across medicinal Asteraceae. In *Atractylodes macrocephala*, transcriptome sequencing of five tissues revealed that multiple unigenes involved in sesquiterpenoid biosynthesis and 287 *CYP450s* were annotated, with *AmFDPS* experimentally verified to catalyze sesquiterpenoid biosynthesis. Similarly, in *Atractylodes lancea*, integrated NGS and SMRT sequencing identified nine full-length terpene synthase genes, among which *AlTPS1* and *AlTPS2* were functionally characterized: *AlTPS1* catalyzes the biosynthesis of elemol, while *AlTPS2* exhibits β-farnesene synthase activity, using FPP as a common substrate [63]. Furthermore, genome editing in *Cichorium intybus* demonstrated that inactivation of the four *CiGAS* genes, encoding germacrene A synthase, almost completely eliminated sesquiterpene lactone accumulation, confirming the essential role of *GAS* in the first committed step of STL biosynthesis [64]. Collectively, these studies illustrate that while *Eupatorium lindleyanum* shares the conserved terpenoid biosynthetic “toolkit” with these other Asteraceae species, the family as a whole deploys lineage-specific structural genes and enzymatic modifications on a conserved sesquiterpenoid scaffold, leading to significant metabolic diversification. Of note, the conversion of germacrene D to eupalinolide B, although chemically plausible, lacks direct metabolomic and WGCNA co-expression support in our data, and is therefore included in Fig 6 as a homology-based inference from other Asteraceae species [65]. Beyond the pathway-level inferences discussed above, the absence of root-dominant modules among the WGCNA-identified candidates merits a methodological clarification.It is noteworthy that although the WGCNA input included DEG Cluster 5 (root-to-flower decreasing trend), none of the seven modules significantly correlated with the selected DSMs displayed a root-predominant pattern. This can be explained by two methodological factors. First, our stepwise strategy employed Mfuzz as a coarse filter to reduce the candidate DEG pool based on trend similarity, followed by WGCNA as a more stringent tool to pinpoint genes with tight module–trait correlations, progressively narrowing the candidate scope. Second, and more critically, among the ten representative sesquiterpenoids used for module–trait correlation analysis, only three exhibited root-predominant accumulation, while the remaining seven were preferentially accumulated in flower. This numerical imbalance inherently biases WGCNA toward detecting modules associated with the dominant aerial-tissue metabolites, diminishing the statistical leverage of root-enriched genes in module identification. Thus, the absence of a root-dominant module does not indicate neglect of root-relevant candidates, but rather reflects the quantitative composition of the metabolite panel and the logic of our stepwise prioritization strategy.

### 4.3. Transcription factors as candidate regulators of sesquiterpenoid biosynthesis

The involvement of transcription factors (TFs) in the regulation of sesquiterpenoid biosynthesis is well documented. In this study, we identified five major TF families—*AP2/ERF*, *NAC*, *WRKY*, *MYB*, and *bHLH*—whose expression levels were significantly correlated with the differential accumulation of ten representative sesquiterpenoids in *Eupatorium lindleyanum*. Among these, the *MYB* family exhibited the highest number of significantly correlated clusters, followed by *bHLH*, *WRKY*, *AP2/ERF*, and *NAC*, suggesting a coordinated, tissue-specific regulatory network. These correlations are consistent with known functions of these families in terpenoid metabolism. For instance, *MYB* and *bHLH* TFs, often forming MBW complexes, are known to activate mevalonate pathway genes (e.g., *HMGCR*, *FDPS*) and terpene synthase genes [66]. *AP2/ERF* TFs have been implicated in directly activating sesquiterpene synthase genes like *GAS* by binding to cis-elements in their promoters, which aligns with the positive correlation observed between specific *AP2/ERFs* and germacrene-type sesquiterpenoids such as eupalinolide B [67]. Similarly, *WRKY* TFs, frequently associated with defense responses, showed strong correlations with compounds like isoalantolactone and shizukanolide H, potentially through the regulation of downstream cytochrome P450 monooxygenases that diversify sesquiterpene skeletons [68]. *NAC* TFs have also been implicated in terpenoid biosynthesis; for instance, *SaNAC30* directly activates the MVA pathway gene *SaMVK* in sandalwood, and *AaNACs* regulate monoterpene synthase genes in kiwifrui [69]. It must be emphasized, however, that this analysis is based on correlation, not causation. While these findings provide a robust framework for prioritizing candidate regulators, experimental validation—such as gene silencing or overexpression assays—is essential to establish direct regulatory relationships [70].

### 4.4. Limitations

A key limitation is the correlative nature of our candidate gene assignments. Although WGCNA has prioritized genes strongly associated with sesquiterpenoid accumulation, these associations do not establish causality. The proposed pathway and regulatory network remain hypotheses requiring functional validation through gene silencing, overexpression, or CRISPR/Cas9 knockout. Likewise, the regulatory roles of transcription factors need direct evidence from promoter-binding assays. Additionally, our tissue-specific profiling provides only a spatial snapshot; temporal and environmental dynamics remain unexplored. Despite these limitations, this study offers a robust foundation and prioritized gene list for future functional characterization toward metabolic engineering of high-yielding *Eupatorium lindleyanum*.

## 5. Conclusion

This study presents a comprehensive metabolomic and transcriptomic analysis of *Eupatorium lindleyanum*, revealing tissue-specific accumulation patterns of 321 sesquiterpenoids across root, stem, leaf, and flower. Integration of metabolite profiling with transcriptome data identified 20 structural genes (88 unigenes) involved in sesquiterpenoid biosynthesis, along with five transcription factor families (*AP2/ERF*, *NAC*, *WRKY*, *MYB*, and *bHLH*) significantly correlated with sesquiterpenoid metabolites accumulation. WGCNA further prioritized key candidate genes and hub regulators within co-expression modules associated with specific sesquiterpenoids. These findings provide a valuable resource for understanding the molecular mechanisms governing tissue-specific sesquiterpenoid biosynthesis in *Eupatorium lindleyanum* and offer potential targets for genetic engineering and breeding programs aimed at enhancing production of bioactive compounds. Future work should focus on functional validation of these candidate genes and transcription factors.

## Supporting information

S1 File Supplementary Figure 1: photographs of different tissues of *Eupatorium lindleyanum*, including root, stem, leaf, and flower. Supplementary Figure 2: cluster heatmap of genes contained in the WGCNA module with strong correlation to sesquiterpene metabolites. Supplementary Figure 3: correlation network diagram of hub genes in the seven WGCNA modules. Supplementary Figure 4: the expression levels of sesquiterpene biosynthesis-related transcription factors in root, stem, leaf, and flower. Supplementary Table 1: list of primers for candidate and reference genes. Supplementary Table 2: sesquiterpenoid metabolites identified in *Eupatorium lindleyanum* by UPLC-MS/MS. Supplementary Table 3: 299 differentially accumulated sesquiterpenoid metabolites (DSMs) identified across pairwise tissue comparisons in *Eupatorium lindleyanum*. Supplementary Table 4: DSMs overlapping across all six pairwise comparisons. Supplementary Table 5: transcriptome data quality control parameters for 12 samples. Supplementary Table 6: all genes annotated based on KEGG, NR, Swissprot, TrEMBL, KOG and GO databases. Supplementary Table 7: differentially expressed genes (DEGs) identified in the six pairwise tissue comparisons of *Eupatorium lindleyanum*. Supplementary Table 8: genes related to sesquiterpenoid biosynthesis in *Eupatorium lindleyanum*.
(ZIP)

## Abbreviations

UPLC‒MS/MS: Ultra performance liquid chromatography‒tandem mass spectrometry
DSMs: Differentially accumulated sesquiterpenoid metabolites
DEGs: Differentially expressed genes
TFs: Transcription factors
MVA: Mevalonate pathway
MEP: 2-C-methyl-D-erythritol 4-phosphate pathway
*GAO*: Germacrene A oxidase
*GAS*: Germacrene A synthase
*PMK*: Phosphomevalonate kinase
*ACAT*: Acetyl-CoA acetyltransferase
*HMGCS*: Hydroxymethylglutaryl-CoA synthase
*HMGCR*: Hydroxymethylglutaryl-CoA reductase
*MVK*: Mevalonate kinase
*MVD*: Diphosphomevalonate decarboxylase
*FDPS*: Farnesyl diphosphate synthase
*IDI*: Isopentenyl-diphosphate Delta-isomerase
*dxs*: 1-deoxy-D-xylulose-5-phosphate synthase
*dxr*: 1-deoxy-D-xylulose-5-phosphate reductoisomerase
*ispD*: 2-C-methyl-D-erythritol 4-phosphate cytidylyltransferase
*ispE*: 4-(cytidine 5’-diphospho)-2-C-methyl-D-erythritol kinase
*ispF*: 2-C-methyl-D-erythritol 2,4-cyclodiphosphate synthase
*ispG*: (E)-4-hydroxy-3-methylbut-2-enyl-diphosphate synthase
*ispH*: 4-hydroxy-3-methylbut-2-en-1-yl diphosphate reductase
*FLDH*: NAD+-dependent farnesol dehydrogenase
NES1: (3S,6E)-nerolidol synthase
*GDS*: Germacrene D synthase
TCM: Traditional chinese medicine
RNA-seq: RNA sequencing
FPKM: Fragments per kilobase of transcript per million mapped reads
PCA: Principal component analysis
OPLS-DA: Orthogonal projections to latent structures-discriminant analysis
VIP: Variable importance in projection
FDR: False discovery rate
GO: Gene ontology
KEGG: Kyoto encyclopedia of genes and genomes
NR: Non-redundant protein sequence database
KOG: euKaryotic Orthologous Groups
qRT-PCR: Quantitative real-time polymerase chain reaction
WGCNA: Weighted Gene Co-Expression Network Analysis
