# Supplementary material for: Multi-omics integration reveals tissue-specific biosynthesis of sesquiterpenoids in the medicinal plant Eupatorium lindleyanum
Source: PLoS One. 2026 Oct 1;21(10):e0359566. doi: 10.1371/journal.pone.0359566 (PMC13630220; doi:10.1371/journal.pone.0359566)
Supplement: Supplementary file 2 [file pone.0359566.s001.zip › Supplementary/Supplementary Figure 3 .docx]

Supplementary Figure 3: Correlation network diagram of hub genes in the seven WGCNA modules.
