# Supplementary material for: Multi-omics integration reveals tissue-specific biosynthesis of sesquiterpenoids in the medicinal plant Eupatorium lindleyanum
Source: PLoS One. 2026 Oct 1;21(10):e0359566. doi: 10.1371/journal.pone.0359566 (PMC13630220; doi:10.1371/journal.pone.0359566)
Supplement: Supplementary file 2 [file pone.0359566.s001.zip › Supplementary/Supplementary Figure 4 .docx]

Supplementary Figure 4: The expression levels of sesquiterpene biosynthesis-related tran

scription factors in roots, stems, leaves, and flowers.
