# Supplementary material for: Multi-omics integration reveals tissue-specific biosynthesis of sesquiterpenoids in the medicinal plant Eupatorium lindleyanum
Source: PLoS One. 2026 Oct 1;21(10):e0359566. doi: 10.1371/journal.pone.0359566 (PMC13630220; doi:10.1371/journal.pone.0359566)
Supplement: Supplementary file 3 [file pone.0359566.s002.docx]

# Comments on the manuscript

## Minor comments

1. Supplementary Figure 1: Add a scale bar (or a ruler in the photo) so tissue size is interpretable.
2. Supplementary Table 1: Add amplicon size (bp) and gene annotation.
3. Supplementary Table 2: Add mass error (ppm), annotation level, database evidence, and retention time.
4. Figure 1B: The legend is unnecessary, since every point is already labeled; remove it and enlarge the panel. Also correct the typo “Stam-3” to “Stem-3.”
5. Figure 3A: Use the same color scheme as Figure 2B and remove the legend.
6. Figures 4E and 5C: Sort the Y-axis categories by Rich Factor, from highest to lowest, to make the most significant terms easier to identify.
7. Figure 6 / lines 421 and 614: CYP71D55 (“premnaspirodiene,” not “prenaspirodiene,” per Takahashi et al.) is characterized in *Hyoscyamus muticus* (Solanaceae), and CDN1 specifically initiates gossypol biosynthesis in cotton (*Gossypium*, Malvaceae) — neither pathway is established in Asteraceae. Please confirm whether solavetivone or gossypol were actually detected among the 321 profiled metabolites; if not, qualify or remove these branches from Figure 6, and correct the enzyme name spelling throughout.
8. Section 3.7: The stated ranking “MYB, followed by bHLH, WRKY, AP2/ERF, and NAC” does not match the reported cluster counts (23, 21, 19, 20, 15) — the correct descending order would place AP2/ERF (20) before WRKY (19). Please correct the order (or the counts, if the order is what is accurate).
9. Sections 2.8/3.8: The Methods and the Results section title both describe the 12 qPCR-validated genes as “candidate genes,” but the Results text calls them “randomly selected genes.” Please clarify which description is correct.
10. Figure 5A: The seven modules are listed in two different orders within the same section (“…yellow, pink, red, turquoise” vs. “…pink, red, turquoise, yellow”), risking miscounted hub-gene attribution. Please use a consistent order throughout.
11. Line 121: Specify the age of the plant/tissue sampled, as this factor can significantly affect tissue-specific differential expression.
12. Line 51 (ref. 6): This reference (“Structural characterization of the plastid genome and comparative mitochondrial genomics of Eupatorium lindleyanum”) concerns organellar genomics, not phytochemistry or compound isolation. Please correct the citation.
13. Line 73 (ref. 15): This reference (Wu et al. 2021, safflower genome) focuses on linoleic acid and flavonoid biosynthesis, not sesquiterpenoids or caryophyllene gene clusters. Please confirm whether it supports this specific claim or should be cited in another context.
14. Line 270: The text reports “146 upregulated and 46 downregulated” metabolites for leaf vs. root, but Figure 2A’s own data show “146, 76” for that comparison. Please verify and correct.
15. Line 299: The phrase “averaged ≥X%” is confusing, since 98.38%/95.35% are actually the minimum values across samples, not the averages. Please reword for clarity.
16. Line 317: The leaf-root and flower-stem DEG comparisons are not reported in the text; please add them or note that they appear only in the supplementary table.
17. Line 304: “31.6% and 23.12%” — inconsistent decimal precision between PC1 and PC2; please standardize.
18. Line 432: The number of metabolites associated with AP2/ERF is not reported (only “20 clusters”), unlike the other four TF families.
19. Line 469: Specify which sesquiterpenoids found in other plant systems overlap with those identified in this study.
20. Line 475: Report the log2FC values in parentheses for the differentially accumulated metabolites mentioned.
21. Lines 479–480: Specify which genes may regulate sesquiterpenoid biosynthesis, rather than referring to them generically.
22. Line 486: Report which of the sesquiterpenoids found in this study are known to be biosynthesized in response to biotic or abiotic elicitation.
23. Line 783 (ref. 41): This reference concerns *Coptidis Rhizoma*, a plant known for berberine-type alkaloids — an unrelated genus and chemical class. Please replace it with a more relevant reference.

## Major comments

1. Supplementary Table 1 contains gene-identity inconsistencies relative to Figure 8. (a) The primer table lists **GGPS** (geranylgeranyl diphosphate synthase, Cluster-28107.0) in the position where Figure 8 and the rest of the manuscript instead validate **GAO** (Cluster-66246.0), a gene that is part of the 22 candidate structural genes; GGPS itself appears nowhere else in the manuscript and is not obviously part of the same pathway (it is typically associated with diterpenoid/carotenoid, not sesquiterpenoid, biosynthesis). (b) *ACAT* is labeled Cluster-13166.0 in Figure 8 but Cluster-56578.0 in Supplementary Table 1. Please verify which unigene IDs and gene identities are correct and correct Supplementary Table 1 accordingly.
2. Section 2.4 states that DAMs — and, by extension, the 299 DSMs underlying the rest of the manuscript — were defined partly by VIP scores from an OPLS-DA model, but no validation of that model (R²Y, Q², or a permutation test) is reported anywhere. Given the supervised, overfitting-prone nature of OPLS-DA, especially with n = 3 replicates per group, this validation is necessary to confirm that the 299 DSMs reflect genuine biological signal rather than model overfitting. *Please report the relevant OPLS-DA validation statistics (R²Y, Q², and/or permutation test results).*
3. The claim that qRT-PCR “showed a consistent trend” with RNA-Seq FPKM values (Section 3.8) is not supported by a quantitative statistic. *Please report a correlation coefficient (e.g., Pearson’s r) between qPCR and FPKM values for each of the 12 validated genes individually.*
4. A single, non-independently-validated reference gene (β-actin) was used for qPCR normalization across tissues as divergent as root and flower. *Per MIQE guidelines, I recommend validating the stability of at least two reference genes (e.g., via geNorm or NormFinder) across this tissue range, or explicitly justifying the use of a single reference gene.*
5. Line 241: The claim of a “gradual increase” in sesquiterpenoid accumulation across tissues is not supported by a statistical test. *Given that three biological replicates are available, I recommend a formal statistical comparison (e.g., ANOVA or Kruskal-Wallis with post-hoc tests) to support this claim.*
6. Line 249: The statement that “inter-group comparisons revealed relatively weak correlations” (Figure 1C) accurately describes the root-vs-rest comparisons but not the stem-leaf, stem-flower, or leaf-flower comparisons, which are in fact very high. *Please qualify this sentence so that it does not generalize to all four tissues a pattern that, in the data, applies only to root.*
7. Clusters 1, 3, and 6 from the DEG expression clustering (Figure 4D) — together ~42% of all DEGs (22,638 of 53,610 genes) — are never characterized in the text or figure legend. *Please briefly describe the expression pattern each cluster represents, even if functional analysis is pursued only for the three clusters that correlate with the DSMs.*
8. The Discussion should be restructured into labeled subsections to match the scale and diversity of the datasets generated (metabolomics, transcriptomics, WGCNA, transcription factor correlations, and qRT-PCR validation), ensuring that each data stream receives proportionate interpretive treatment and that the study’s limitations are addressed explicitly. I suggest:

**4.1 Tissue-specific accumulation of sesquiterpenoids and its transcriptional basis** — consolidating the discussion of the 321 metabolites, 299 DSMs, and their associated DEG clusters.

**4.2 Integration of transcriptomic and metabolomic data: structural genes underlying the biosynthetic pathway** — consolidating the discussion of the 22 candidate structural genes and their comparison with other Asteraceae. This subsection should specifically address: (a) why none of the seven WGCNA modules significantly correlated with the selected DSMs shows a root-predominant expression pattern, despite root being one of the three principal tissue-specific patterns established earlier in the manuscript (this negative result is currently not discussed anywhere); and (b) which branches of the proposed pathway (Figure 6) are supported by the study’s own metabolomic evidence versus inferred by homology to pathways characterized in taxonomically distant species (e.g., the solavetivone branch from *Hyoscyamus muticus* and the gossypol branch from *Gossypium*; see comment 7).

**4.3 Transcription factors as candidate regulators of sesquiterpenoid biosynthesis** — consolidating the discussion of the five TF families and their correlation with the ten selected metabolites.

**4.4 Limitations** — a new subsection addressing, at minimum: (i) the modest sample size (n = 3 per tissue) and the absence of OPLS-DA validation (see comment 25); (ii) reliance on a single, non-validated qPCR reference gene (see comment 27); and (iii) the correlative, non-causal nature of all candidate gene and transcription factor assignments, pending functional validation (e.g., gene silencing/overexpression).
