# Supplementary material for: Multi-omics integration reveals tissue-specific biosynthesis of sesquiterpenoids in the medicinal plant Eupatorium lindleyanum
Source: PLoS One. 2026 Oct 1;21(10):e0359566. doi: 10.1371/journal.pone.0359566 (PMC13630220; doi:10.1371/journal.pone.0359566)
Supplement: Peer review history

# Peer review: Decision Letter 0

**DOI:** 10.1371/journal.pone.0359566.r001

**Authors:** Rongchun Han

5 Aug 2026

Dear Dr. Wang,

Thank you for submitting your manuscript to PLOS ONE. After careful consideration, we feel that it has merit but does not fully meet PLOS ONE’s publication criteria as it currently stands. Therefore, we invite you to submit a revised version of the manuscript that addresses the points raised during the review process.

Please submit your revised manuscript by Oct 04 2026 11:59PM. If you will need more time than this to complete your revisions, please reply to this message or contact the journal office at plosone@plos.org. When you're ready to submit your revision, log on to https://www.editorialmanager.com/pone/ and select the 'Submissions Needing Revision' folder to locate your manuscript file.

- A letter that responds to each point raised by the academic editor and reviewer(s). You should upload this letter as a separate file labeled 'Response to Reviewers'.
- A marked-up copy of your manuscript that highlights changes made to the original version. You should upload this as a separate file labeled 'Revised Manuscript with Track Changes'.
- An unmarked version of your revised paper without tracked changes. You should upload this as a separate file labeled 'Manuscript'.

If you would like to make changes to your financial disclosure, please include your updated statement in your cover letter. Guidelines for resubmitting your figure files are available below the reviewer comments at the end of this letter.

If applicable, we recommend that you deposit your laboratory protocols in protocols.io to enhance the reproducibility of your results. Protocols.io assigns your protocol its own identifier (DOI) so that it can be cited independently in the future. For instructions see: https://journals.plos.org/plosone/s/submission-guidelines#loc-laboratory-protocols. Additionally, PLOS ONE offers an option for publishing peer-reviewed Lab Protocol articles, which describe protocols hosted on protocols.io. Read more information on sharing protocols at https://plos.org/protocols?utm_medium=editorial-email&utm_source=authorletters&utm_campaign=protocols.

As the corresponding author, your ORCID iD is verified in the submission system and will appear in the published article. PLOS supports the use of ORCID, and we encourage all coauthors to register for an ORCID iD and use it as well. Please encourage your coauthors to verify their ORCID iD within the submission system before final acceptance, as unverified ORCID iDs will not appear in the published article. *Only*  the individual author can complete the verification step; PLOS staff *cannot*  verify ORCID iDs on behalf of authors.

We look forward to receiving your revised manuscript.

Kind regards,

Rongchun Han

Academic Editor

PLOS One

Journal Requirements:

1.Please ensure that your manuscript meets PLOS ONE's style requirements, including those for file naming. The PLOS ONE style templates can be found at

https://journals.plos.org/plosone/s/file?id=wjVg/PLOSOne_formatting_sample_main_body.pdf and

https://journals.plos.org/plosone/s/file?id=ba62/PLOSOne_formatting_sample_title_authors_affiliations.pdf

2. Please note that PLOS One has specific guidelines on code sharing for submissions in which author-generated code underpins the findings in the manuscript. In these cases, we expect all author-generated code to be made available without restrictions upon publication of the work. Please review our guidelines at https://journals.plos.org/plosone/s/materials-and-software-sharing#loc-sharing-code and ensure that your code is shared in a way that follows best practice and facilitates reproducibility and reuse.

3. Please note that funding information should not appear in any section or other areas of your manuscript. We will only publish funding information present in the Funding Statement section of the online submission form. Please remove any funding-related text from the manuscript.

4. We note that the grant information you provided in the ‘Funding Information’ and ‘Financial Disclosure’ sections do not match.

When you resubmit, please ensure that you provide the correct grant numbers for the awards you received for your study in the ‘Funding Information’ section.

5. When completing the data availability statement of the submission form, you indicated that you will make your data available on acceptance. We strongly recommend all authors decide on a data sharing plan before acceptance, as the process can be lengthy and hold up publication timelines. Please note that, though access restrictions are acceptable now, your entire data will need to be made freely accessible if your manuscript is accepted for publication. This policy applies to all data except where public deposition would breach compliance with the protocol approved by your research ethics board. If you are unable to adhere to our open data policy, please kindly revise your statement to explain your reasoning and we will seek the editor's input on an exemption. Please be assured that, once you have provided your new statement, the assessment of your exemption will not hold up the peer review process.

6. PLOS requires an ORCID iD for the corresponding author in Editorial Manager on papers submitted after December 6th, 2016. Please ensure that you have an ORCID iD and that it is validated in Editorial Manager. To do this, go to ‘Update my Information’ (in the upper left-hand corner of the main menu), and click on the Fetch/Validate link next to the ORCID field. This will take you to the ORCID site and allow you to create a new iD or authenticate a pre-existing iD in Editorial Manager.

7. Please include captions for your Supporting Information files at the end of your manuscript, and update any in-text citations to match accordingly. Please see our Supporting Information guidelines for more information: http://journals.plos.org/plosone/s/supporting-information.

8. If the reviewer comments include a recommendation to cite specific previously published works, please review and evaluate these publications to determine whether they are relevant and should be cited. There is no requirement to cite these works unless the editor has indicated otherwise.

Reviewers' comments:

Reviewer's Responses to Questions

**Comments to the Author**

1. Is the manuscript technically sound, and do the data support the conclusions?

Reviewer #1: Yes

Reviewer #2: Yes

**********

2. Has the statistical analysis been performed appropriately and rigorously?

Reviewer #1: Yes

Reviewer #2: Yes

**********

3. Have the authors made all data underlying the findings in their manuscript fully available?

The PLOS Data policy

Reviewer #1: Yes

Reviewer #2: Yes

**********

4. Is the manuscript presented in an intelligible fashion and written in standard English?

Reviewer #1: Yes

Reviewer #2: Yes

**********

Reviewer #1: The paper is written well, acceptable with some minor revise.

1. Just refer to the type of your cDNA sequencing library, sranded or unstranded?

2. Explain in the text that why you didn't quantified and qualified volatile sesquiterpenes with GC-FID and GC-MS.

3. Add your metabolite dataset, tables and identified compounds in a open platforms like figshare.

Reviewer #2: I included an attached Word document titled "Comments for article" where I explain all my comments about the article.

This manuscript addresses a genuine, previously unexplored question: the genetic basis of tissue-specific sesquiterpenoid biosynthesis in Eupatorium lindleyanum. Its main strength is the scale and internal consistency of the multi-omics dataset — 321 sesquiterpenoids profiled across four tissues, 99.43 Gb of RNA-Seq data, and 22 candidate biosynthetic genes — figures we cross-checked throughout this review and found to be traceable and coherent across the Abstract, Results, and Methods. The integrative pipeline (Mfuzz clustering, WGCNA, and independent qRT-PCR validation) is methodologically sound, grounded in well-established terpenoid biochemistry, and appropriately careful to frame gene-metabolite associations as correlative rather than causal. The resulting candidate gene and transcription factor list is a genuinely useful, prioritized resource for future functional validation in this species. Our detailed comments below aim to strengthen the manuscript's rigor and clarity, not to question the value of its underlying dataset or research question

**********

what does this mean?). If published, this will include your full peer review and any attached files.

If you choose “no”, your identity will remain anonymous but your review may still be made public.

**Do you want your identity to be public for this peer review?**  For information about this choice, including consent withdrawal, please see our Privacy Policy

Reviewer #1: No

Reviewer #2: **Yes:**  Olmedo Jesus Cuaspud Caliz

**********

[NOTE: If reviewer comments were submitted as an attachment file, they will be attached to this email and accessible via the submission site. Please log into your account, locate the manuscript record, and check for the action link "View Attachments". If this link does not appear, there are no attachment files.]

To ensure your figures meet our technical requirements, please review our figure guidelines: https://journals.plos.org/plosone/s/figures

You may also use PLOS’s free figure tool, NAAS, to help you prepare publication quality figures: https://journals.plos.org/plosone/s/figures#loc-tools-for-figure-preparation.

NAAS will assess whether your figures meet our technical requirements by comparing each figure against our figure specifications.

Attachment Submitted filename: Comments for article.docx

# Author comment: Author response to Decision Letter 1

**DOI:** 10.1371/journal.pone.0359566.r002

27 Aug 2026

Point-by-Point Response to Reviewer 1

1. Just refer to the type of your cDNA sequencing library, sranded or unstranded?

Response: We thank the reviewer for this highly professional question. We acknowledge that stranded RNA-seq offers certain advantages in specific applications. In our study, the cDNA libraries were constructed using the standard non-stranded (unstranded) double-stranded cDNA synthesis protocol. We have now explicitly stated this information in the Methods 2.5 section of the revised manuscript (Lines 175-177). We recognize that this is a technical limitation.

We would like to clarify that the use of unstranded libraries does not materially affect the main conclusions of our study. All our key analyses—including gene-level expression quantification, WGCNA, and metabolome-transcriptome integration—are based on gene-level abundance, which does not require strand orientation information. While strand-specific data can offer higher resolution for distinguishing overlapping transcripts, our study does not involve analyses that depend on strand specificity, such as antisense transcript detection, non-coding RNA annotation, or promoter-associated RNA analysis. Therefore, we are confident that the unstranded library type is appropriate for the objectives of this study and does not compromise the validity of our findings.

2. Explain in the text that why you didn't quantified and qualified volatile sesquiterpenes with GC-FID and GC-MS.

Response: We sincerely thank the reviewer for this valuable and professional comment. In response, we have added a brief explanation at the end of Section 2.2 (Plant sample pretreatment and metabolite extraction procedures, lines 134-137) to clarify our methodological choice. Although most sesquiterpenes are volatile in nature, their detection by UPLC-MS does not rely on vaporization but on solubility, which is equally valid. Importantly, many of our target compounds are thermally labile (e.g., sesquiterpene lactones) and exist as high-polarity glycosides or acids, which are unsuitable for GC analysis due to decomposition or high boiling points. Therefore, UPLC-MS was deliberately chosen to detect both the volatile aglycones and their polar derivatives under a single, mild chromatographic condition, providing more comprehensive and degradation-free quantitative data than GC-based methods.

3. Add your metabolite dataset, tables and identified compounds in a open platforms like figshare.

Response: We appreciate the reviewer's constructive suggestion. As recommended, we have now deposited our metabolite dataset, including all tables and identified compounds, in the open-access platform SCIENCE DB. The data can be accessed at https://www.scidb.cn/anonymous/ZVlKbmFp. Correspondingly, we have added a Data Availability Statement at the end of the manuscript to indicate this information (lines 712-713).

Point-by-Point Response to Reviewer 2

Minor comments

1. Supplementary Figure 1: Add a scale bar (or a ruler in the photo) so tissue size is interpretable.

Response: We thank the reviewer for this suggestion. We have now added scale bars to each individual panel in Supplementary Figure 1. Because different tissue sections of Eupatorium lindleyanum were imaged at different magnifications, we have included separate scale bars for each subpanel to ensure accurate size interpretation.

2. Supplementary Table 1: Add amplicon size (bp) and gene annotation.

Response: We greatly appreciate the reviewer's insightful and constructive suggestions. In response, we have supplemented Supplementary Table 1 with two additional columns, namely "Gene Annotation" and "Amplicon Size (bp)" (inserted as Columns 2 and 6, respectively), to ensure that all essential information regarding the qPCR primers is fully presented.

3. Supplementary Table 2: Add mass error (ppm), annotation level, database evidence, and retention time.

We thank the reviewer for the constructive suggestion. In response, we have added mass error (ppm), annotation level, and database evidence in columns C, D, and E of Supplementary Table 2, respectively. Regarding the retention time, we regret that we are unable to provide this information. The metabolomics analysis was conducted in collaboration with a third-party service provider, and despite our sincere efforts to obtain the retention time data, they declined to disclose it due to commercial confidentiality. We hope the reviewer can kindly understand this constraint. We appreciate your understanding and valuable input.

4. Figure 1B: The legend is unnecessary, since every point is already labeled; remove it and enlarge the panel. Also correct the typo “Stam-3” to “Stem-3.”

Response: We thank the reviewer for pointing out these oversights in our work. In response, we have removed the legend from Figure 1B, as each data point is already clearly labeled, and have appropriately enlarged the panel to improve readability. The typographical error "Stam-3" has also been corrected to "Stem-3" in the figure (lines 273).

5. Figure 3A: Use the same color scheme as Figure 2B and remove the legend.

Response:We thank the reviewer for the suggestion. We have revised Figure 3A by adopting the same color scheme as used in Figure 2B and have removed the legend accordingly (lines 327).

6. Figures 4E and 5C: Sort the Y-axis categories by Rich Factor, from highest to lowest, to make the most significant terms easier to identify.

Response:We thank the reviewer for this helpful suggestion. As recommended, we have revised Figure 4E by sorting the Y-axis categories in descending order of Rich Factor to highlight the most significant terms. Regarding Figure 5C, we attempted to apply the same sorting procedure; however, we found that after reordering, several key metabolic pathways—namely, metabolic pathways (ko01100), biosynthesis of secondary metabolites (ko01110), sesquiterpenoid and triterpenoid biosynthesis (ko00909), and terpenoid backbone biosynthesis (ko00900)—could no longer be fully displayed in the figure due to space constraints. Given that these pathways are critically relevant to our study, we respectfully request to retain the original layout of Figure 5C. We hope the reviewer can kindly understand this practical limitation. We greatly appreciate your understanding and valuable feedback.

7. Figure 6 / lines 421 and 614: CYP71D55 (“premnaspirodiene,” not “prenaspirodiene,” per Takahashi et al.) is characterized in Hyoscyamus muticus (Solanaceae), and CDN1 specifically initiates gossypol biosynthesis in cotton (Gossypium, Malvaceae) — neither pathway is established in Asteraceae. Please confirm whether solavetivone or gossypol were actually detected among the 321 profiled metabolites; if not, qualify or remove these branches from Figure 6, and correct the enzyme name spelling throughout.

Response: We sincerely thank the reviewer for pointing out this critical issue and for providing the relevant literature reference. Upon careful verification, we fully agree with the reviewer's assessment that neither CYP71D55 nor CDN1 has been functionally characterized in Asteraceae, nor have solavetivone or gossypol been confirmed as biosynthetic products in this family. Accordingly, we have removed both genes and their associated pathway branches from Figure 6. In addition, we have revised the corresponding description throughout the manuscript: the statement "22 structural genes (91 unigenes)" has been corrected to "20 structural genes (88 unigenes)" to reflect this change. We greatly appreciate the reviewer's valuable and constructive comments, which have helped us rectify an oversight in our work. We are grateful for your guidance and understanding.

8. Section 3.7: The stated ranking “MYB, followed by bHLH, WRKY, AP2/ERF, and NAC” does not match the reported cluster counts (23, 21, 19, 20, 15) — the correct descending order would place AP2/ERF (20) before WRKY (19). Please correct the order (or the counts, if the order is what is accurate).

Response: We thank the reviewer for carefully identifying this inconsistency. Upon rechecking our data, we confirm that the reviewer is correct. We have now revised the order in Section 3.7 to reflect the correct descending ranking based on the cluster counts, which should be: MYB (23), followed by bHLH (21), AP2/ERF (20), WRKY (19), and NAC (15). The corresponding text has been amended accordingly (line 452). We appreciate the reviewer's meticulous attention to detail and constructive suggestion.

9. Sections 2.8/3.8: The Methods and the Results section title both describe the 12 qPCR-validated genes as “candidate genes,” but the Results text calls them “randomly selected genes.” Please clarify which description is correct.

Response:We thank the reviewer for identifying this inconsistency. Initially, we intended to randomly select genes for qPCR validation of the transcriptome data. However, we subsequently chose 12 genes from the 20 candidate genes for validation. Due to our oversight, the wording in the Methods and Results sections was not aligned. We have now carefully revised the manuscript and uniformly changed the description to "candidate genes" throughout both sections. We apologize for this error and appreciate the reviewer's careful reading and constructive suggestion.

10. Figure 5A: The seven modules are listed in two different orders within the same section (“…yellow, pink, red, turquoise” vs. “…pink, red, turquoise, yellow”), risking miscounted hub-gene attribution. Please use a consistent order throughout.

Response:We thank the reviewer for pointing out this inconsistency. We have carefully revised Section 3.5 to ensure that the seven modules are consistently listed in the same order throughout—namely, yellow, pink, red, and turquoise (line 385). We have also thoroughly double-checked the hub-gene counts corresponding to each module to ensure accuracy. We appreciate the reviewer's meticulous attention to detail and constructive suggestion.

11. Line 121: Specify the age of the plant/tissue sampled, as this factor can significantly affect tissue-specific differential expression.

Response: We thank the reviewer for this valuable suggestion. In response, we have now specified the age and developmental stage of the sampled plants in Section 2.1 (line 115) of the revised manuscript. Specifically, we have added that the plants were 17-month-old biennials and at the full-flowering stage at the time of tissue collection.

12. Line 51 (ref. 6): This reference (“Structural characterization of the plastid genome and comparative mitochondrial genomics of Eupatorium lindleyanum”) concerns organellar genomics, not phytochemistry or compound isolation. Please correct the citation.

Response:We thank the reviewer for carefully identifying this citation error. We sincerely apologize for our negligence during the literature retrieval process, which led to the inappropriate citation of an organellar genomics reference in the context of phytochemical isolation. We have now corrected this mistake by replacing the original reference with the appropriate one that accurately supports the statement regarding compound isolation from Eupatorium lindleyanum. The revised reference has been updated as reference [6] in the manuscript.

13. Line 73 (ref. 15): This reference (Wu et al. 2021, safflower genome) focuses on linoleic acid and flavonoid biosynthesis, not sesquiterpenoids or caryophyllene gene clusters. Please confirm whether it supports this specific claim or should be cited in another context.

Response: We thank the reviewer for carefully identifying this citation error. We sincerely apologize for our oversight during the reference compilation process, which led to the inappropriate citation of Wu et al. (2021) in the context of sesquiterpenoid biosynthesis. The claim regarding comparative genomic analyses of Silybum marianum and Carthamus tinctorius is in fact supported by reference 14. Reference 15 was inadvertently added alongside it and has now been removed, with all subsequent references renumbered accordingly in the revised manuscript.

14. Line 270: The text reports “146 upregulated and 46 downregulated” metabolites for leaf vs. root, but Figure 2A’s own data show “146, 76” for that comparison. Please verify and correct.

Response: We thank the reviewer for catching this error. We apologize for the writing mistake. The correct numbers are 146 upregulated and 76 downregulated metabolites for the leaf vs. root comparison, and this has now been corrected in the revised manuscript (line 286).

15. Line 299: The phrase “averaged ≥X%” is confusing, since 98.38%/95.35% are actually the minimum values across samples, not the averages. Please reword for clarity.

Response:We thank the reviewer for the careful observation. We agree that the original wording was imprecise. The sentence has now been revised to: "The Q20 and Q30 values across all samples were ≥98.38% and ≥95.35%, respectively, with GC content ranging from 42.79% to 44.1%." This correction has been made in the revised manuscript (lines 315-316).

16. Line 317: The leaf-root and flower-stem DEG comparisons are not reported in the text; please add them or note that they appear only in the supplementary table.

Response: We thank the reviewer for pointing out this omission. We have now supplemented the numbers for the leaf-root and flower-stem DEG comparisons in Section 3.4 of the revised manuscript, including both the upregulated and downregulated counts. The corresponding data have also been added to the text accordingly (lines 340-342).

17. Line 304: “31.6% and 23.12%” — inconsistent decimal precision between PC1 and PC2; please standardize.

Response:We thank the reviewer for noticing this inconsistency. We have now standardized the decimal precision by changing "31.6%" to "31.60%" in the revised manuscript to ensure consistency with "23.12%." (line 320)

18. Line 432: The number of metabolites associated with AP2/ERF is not reported (only “20 clusters”), unlike the other four TF families.

Response: We apologize for this writing oversight. The number of metabolites associated with the AP2/ERF transcription factor family was inadvertently omitted. We have now revised the text to "AP2/ERF (20 clusters with 10 metabolites)" to maintain consistency with the other four TF families. The correction has been made in the revised manuscript (line 452).

19. Line 469: Specify which sesquiterpenoids found in other plant systems overlap with those identified in this study.

Response: We thank the reviewer for this valuable suggestion. In response, we have revised the description in Section 4.1 of the revised manuscript to specify the sesquiterpenoids that overlap with those previously reported in other plant systems (lines 498-501). Specifically, we have listed compounds including eupalinolide B, zaluzanin C, isoalantolactone, dehydrocostus lactone, costunolide, spathulenol, and atractylenolide I/II, all of which have been previously characterized in other Asteraceae species and show consistent profiles with our findings in Eupatorium lindleyanum.

20. Line 475: Report the log2FC values in parentheses for the differentially accumulated metabolites mentioned.

Response: We thank the reviewer for this valuable suggestion. In response, we have now added the Log2FC values in parentheses for all the differentially accumulated metabolites mentioned in the text (Agalleudesmanol D, Calacorene oxide, Curcumafuranol, 13-Nor-desacylcynaropicrin, and Oxyphyllenone A) in the revised manuscript (lines 504-511).

21. Lines 479–480: Specify which genes may regulate sesquiterpenoid biosynthesis, rather than referring to them generically.

Response: We thank the reviewer for this insightful comment. We agree that the original statement was indeed vague. The reason is that this paragraph focuses on the differential accumulation of metabolites, and we only briefly mention the possibility of gene regulation here, with a more detailed discussion of the specific regulatory genes presented in the subsequent section. To address this concern, we have revised the sentence to clearly indicate that this question is further investigated in the following integrative analysis. See the lines 513-515 of the revised manuscript for details.

22. Line 486: Report which of the sesquiterpenoids found in this study are known to be biosynthesized in response to biotic or abiotic elicitation.

Response: We thank the reviewer for this suggestion. We have revised lines 523-527 to specify several sesquiterpenoidsthat are known to accumulate in response to biotic or abiotic elicitation in Asteraceae species, with relevant citations added.

23. Line 783 (ref. 41): This reference concerns Coptidis Rhizoma, a plant known for berberine-type alkaloids — an unrelated genus and chemical class. Please replace it with a more relevant reference.

Response: We thank the reviewer for this suggestion. We have replaced the original reference 41 (on Coptidis Rhizoma and berberine-type alkaloids) with a more relevant citation that accurately corresponds to the chemical class discussed in our study. The citation in the text has been updated accordingly, and the reference list has been renumbered. This reference is now listed as reference 40 in the revised manuscript.

Major comments

1. Supplementary Table 1 contains gene-identity inconsistencies relative to Figure 8. (a) The primer table lists GGPS (geranylgeranyl diphosphate synthase, Cluster-28107.0) in the position where Figure 8 and the rest of the manuscript instead validate GAO (Cluster-66246.0), a gene that is part of the 22 candidate structural genes; GGPS itself appears nowhere else in the manuscript and is not obviously part of the same pathway (it is typically associated with diterpenoid/carotenoid, not sesquiterpenoid, biosynthesis). (b) ACAT is labeled Cluster-13166.0 in Figure 8 but Cluster-56578.0 in Supplementary Table 1. Please verify which unigene IDs and gene identities are correct and correct Supplementary Table 1 accordingly.

Response:We thank the reviewer for their careful and thorough review of our data. We have carefully re-examined the relevant materials and acknowledge that errors were introduced during the preparation of Supplementary Table 1 due to inadvertent copy-paste mistakes.

Specifically, we have made the following corrections:

(a) The entry previously listed as GGPS (geranylgeranyl diphosphate synthase, Cluster-28107.0) has been replaced with GAO (Cluster-66246.0), which is the correct gene validated in Figure 8 and included among the 20 candidate structural genes. As the reviewer correctly noted, GGPS is not part of the sesquiterpenoid biosynthesis pathway and does not appear elsewhere in the manuscript.

(b) The unigene ID for ACAT has been corrected from Cluster-56578.0 to Cluster-13166.0, aligning Supplementary Table 1 with Figure 8.

All corrections have been made in the revised Supplementary Table 1, which now consistently matches Figure 8 and the main text. We apologize for these oversights and are grateful to the reviewer for bringing them to our attention.

2. Section 2.4 states that DAMs — and, by extension, the 299 DSMs underlying the rest of the manuscript — were defined partly by VIP scores from an OPLS-DA model, but no validation of that model (R²Y, Q², or a permutation test) is reported anywhere. Given the supervised, overfitting-prone nature of OPLS-DA, especially with n = 3 replicates per group, this validation is necessary to confirm that the 299 DSMs reflect genuine biological signal rather than model overfitting. Please report the relevant OPLS-DA validation statistics (R²Y, Q², and/or permutation test results).

Response: We thank the reviewer for this important suggestion. In the revised manuscript, we have added the OPLS-DA validation statistics in Section 2.4 and Supplementary Table 3. For all six pairwise comparisons, the Q² values ranged from 0.992 to 0.995, R²X from 0.804 to 0.860, and R²Y was 1.0 for all models. These high Q² values and the minimal gap between R²Y and Q² confirm that the models are robust and not overfitted (lines 157-161).

3. The claim that qRT-PCR “showed a consistent trend” with RNA-Seq FPKM values (Section 3.8) is not supported by a quantitative statistic. Please report a correlation coefficient (e.g., Pearson’s r) between qPCR and FPKM values for each of the 12 validated genes individually.

Response: We thank the reviewer for this rigorous and constructive suggestion, which has helped us improve the robustness of our manuscript. In the revised manuscript, we have performed Pearson correlation analysis between the qRT-PCR expression levels and the corresponding RNA-Seq FPKM values for each of the 12 candidate genes across the four examined tissues (root, stem, leaf, and flower). The correlation coefficients (r) and associated p-values for all 12 genes have now been incorporated into Section 3.8 of the revised manuscript (lines 469-476).

4. A single, non-independently-validated reference gene (β-actin) was used for qPCR normalization across tissues as divergent as root and flower. Per MIQE guidelines, I recommend validating the stability of at least two reference genes (e.g., via geNorm or NormFinder) across this tissue range, or explicitly justifying the use of a single reference gene.

Response: We sincerely thank the reviewer for this valuable and professional suggestion. We completely agree that validating the stability of reference genes is essential for accurate qRT-PCR normalization, especially across distinct tissues such as root and flower. In our previous studies, we had already evaluated the expression stability of β-actin across different tissues of Eupatorium lindleyanum, and the results were published elsewhere. However, we regret that this information was inadvertently omitted from the present manuscript. In the revised version, we have now incorporated the stability parameters of β-actin in Section 2.8 (lines 234-239). Specifically, we assessed its stability using geNorm (M = 0.527), NormFinder (stability value = 0.082), and BestKeeper (SD = 0.453), all of which fall within the accepted thresholds, confirming that β-actin exhibits good expression stability and is suitable as a reference gene for qRT-PCR normalization in this study. We are grateful for the reviewer's careful reading and constructive comments, which have helped improve the rigor of our work.

5. Line 241: The claim of a “gradual increase” in sesquiterpenoid accumulation across tissues is not supported by a statistical test. Given that three biological replicates are available, I recommend a formal statistical comparison (e.g., ANOVA or Kruskal-Wallis with post-hoc tests) to support this claim.

Response: We thank the reviewer for this suggestion. In fact, we had already performed a one-way ANOVA on the data presented in Figure 1A, and the significant differences among tissues are indicated by different lowercase letters above the bars (P < 0.05). We apologize for not making this clear in the figure legend. We have now revised the legend to explicitly state: "Different letters above the bars indicate significant differences between tissues (P < 0.05)." We hope this clarification addresses the reviewer's concern.

6. Line 249: The statement that “inter-group comparisons revealed relatively weak correlations” (Figure 1C) accurately describes the root-vs-rest comparisons but not the stem-leaf, stem-flower, or leaf-flower comparisons, which are in fact very high. Please qualify this sentence so that it does not generalize to all four tissues a pattern that, in the data, applies only to root.

Response: We thank the reviewer for this valuable suggestion. We have revised the description in Section 3.1 to more accurately reflect the data, specifying that weak correlations were observed between root and the above-ground tissues, while correlations among stem, leaf, and flower were relatively strong (lines 261-265). The revised sentence now reads: "In contrast, inter-group comparisons revealed that root exhibited relatively weak correlations with stem, leaf, and flower, whereas correlations among the above-ground tissues (stem, leaf, and flower) were relatively strong. This suggests a clear metabolic distinction between root and above-ground tissues, while the metabolic profiles of stem, leaf, and flower are more similar to one another." We hope this revision adequately addresses the reviewer's concern.

7. Clusters 1, 3, and 6 from the DEG expression clustering (Figure 4D) — together ~42% of all DEGs (22,638 of 53,610 genes) — are never characterized in the text or figure legend. Please briefly describe the expression pattern each cluster represents, even if functional analysis is pursued only for the three clusters that correlate with the DSMs.

Response: We thank the reviewer for this suggestion. We have now added a brief description of the expression patterns of Clusters 1, 3, and 6 in Section 3.4 to provide a more comprehensive characterization of all six clusters (lines 347-352). Specifically, we have described that genes in Cluster 1 showed relatively high expression in stem, Cluster 3 exhibited a gradually increasing expression trend across root, stem, leaf, and flower, and Cluster 6 displayed relatively high expression in root. We believe this addition makes the description of the clustering results more complete and addresses the reviewer's concern.

8. The Discussion should be restructured into labeled subsections to match the scale and diversity of the datasets generated (metabolomics, transcriptomics, WGCNA, transcription factor correlations, and qRT-PCR validation), ensuring that each data stream receives proportionate interpretive treatment and that the study’s limitations are addressed explicitly. I suggest:

4.1 Tissue-specific accumulation of sesquiterpenoids and its transcriptional basis — consolidating the discussion of the 321 metabolites, 299 DSMs, and their associated DEG clusters.

4.2 Integration of transcriptomic and metabolomic data: structural genes underlying the biosynthetic pathway — consolidating the discussion of the 22 candidate structural genes and their comparison with other Asteraceae. This subsection should specifically address: (a) why none of the seven WGCNA modules significantly correlated with the selected DSMs shows a root-predominant expression pattern, despite root being one of the three principal tissue-specific patterns established earlier in the manuscript (this negative result is currently not discussed anywhere); and (b) which branches of the proposed pathway (Figure 6) are supported by the study’s own metabolomic evidence versus inferred by homology to pathways characterized in taxonomically distant species (e.g., the solavetivone branch from Hyoscyamus muticus and the gossypol branch from Gossypium; see comment 7).

4.3 Transcription factors as candidate regulators of sesquiterpenoid biosynthesis — consolidating the discussion of the five TF families and their correlation with the ten selected metabolites.

4.4 Limitations — a new subsection addressing, at minimum: (i) the modest sample size (n = 3 per tissue) and the absence of OPLS-DA validation (see comment 25); (ii) reliance on a single, non-validated qPCR reference gene (see comment 27); and (iii) the correlative, non-causal nature of all candidate gene and transcription factor assignments, pending functional validation (e.g., gene silencing/overexpression).

Response: We thank the reviewer for this constructive suggestion. In accordance with your advice, we have restructured the Discussion section into four labeled subsections to better reflect the scale and diversity of the datasets generated in this study.

Specifically, Section 4.1 has been reorganized as "Tissue-specific accumulation of sesquiterpenoids and its transcriptional basis," which consolidates the discussion of the 321 identified metabolites, 299 DSMs, and their associated DEG clusters. Section 4.2 is now titled "Integration of transcriptomic and metabolomic data: structural genes underlying the biosynthetic pathway." In this subsection, we have specifically addressed: (a) the absence of root-predominant expression patterns among the seven WGCNA modules significantly correlated with the selected DSMs, and (b) which branches of the proposed pathway (Figure 6) are supported by our own metabolomic evidence versus those inferred by homology from pathways characterized in taxonomically distant species. Section 4.3 has been revised as "Transcription factors as candidate regulators of sesquiterpenoid biosynthesis," covering the five TF families and their correlations with the ten selected metabolites. Finally, Section 4.4 has been added to discuss the limitations of the study.

Regarding the limitations, we have focused our discussion on the correlative and non-causal nature of our candidate gene and transcription factor assignments, which await functional validation (e.g., via gene silencing or overexpression). We would like to note that the other two issues raised by the reviewer—OPLS-DA validation and qPCR reference gene stability—have already been addressed in Section 2.4 and Section 2.8, respectively; therefore, we have not reiterated them as limitations in Section 4.4.

We believe these revisions have made the Discussion more structured and comprehensive, and we are grateful for the reviewer's valuable guidance.

Attachment Submitted filename: Response to Reviewers.pdf

# Peer review: Decision Letter 1

**DOI:** 10.1371/journal.pone.0359566.r003

**Authors:** Rongchun Han

15 Sep 2026

<p>Multi-omics integration reveals tissue-specific biosynthesis of sesquiterpenoids in the medicinal plant Eupatorium lindleyanum

PONE-D-26-17903R1

We’re pleased to inform you that your manuscript has been judged scientifically suitable for publication and will be formally accepted for publication once it meets all outstanding technical requirements.

Within one week, you’ll receive an e-mail detailing the required amendments. When these have been addressed, you’ll receive a formal acceptance letter and your manuscript will be scheduled for publication.

An invoice will be generated when your article is formally accepted. Please note, if your institution has a publishing partnership with PLOS and your article meets the relevant criteria, all or part of your publication costs will be covered. Please make sure your user information is up-to-date by logging into Editorial Manager at Editorial Manager® and clicking the ‘Update My Information' link at the top of the page. For questions related to billing, please contact billing support.

If your institution or institutions have a press office, please notify them about your upcoming paper to help maximize its impact. If they’ll be preparing press materials, please inform our press team as soon as possible -- no later than 48 hours after receiving the formal acceptance. Your manuscript will remain under strict press embargo until 2 pm Eastern Time on the date of publication. For more information, please contact onepress@plos.org.

Kind regards,

Rongchun Han

Academic Editor

PLOS One

Reviewers' comments:

Reviewer's Responses to Questions

**Comments to the Author**

Reviewer #1: All comments have been addressed

Reviewer #2: All comments have been addressed

**********

2. Is the manuscript technically sound, and do the data support the conclusions?

Reviewer #1: (No Response)

Reviewer #2: Yes

**********

3. Has the statistical analysis been performed appropriately and rigorously?

Reviewer #1: Yes

Reviewer #2: Yes

**********

4. Have the authors made all data underlying the findings in their manuscript fully available?

The PLOS Data policy

Reviewer #1: Yes

Reviewer #2: Yes

**********

5. Is the manuscript presented in an intelligible fashion and written in standard English?

Reviewer #1: Yes

Reviewer #2: Yes

**********

Reviewer #1: The authors have carried out a meaningful work. All amendments are done and now it can be accepted in this form.

Reviewer #2: Overall, the authors have substantially and rigorously addressed the concerns raised in the initial review. The revised manuscript now includes appropriate statistical validation of the multivariate models, a properly validated qPCR reference gene, quantitative correlation statistics between transcriptomic and qPCR data, and a corrected, taxonomically consistent biosynthetic pathway. The Discussion has also been reorganized to better reflect the scope and depth of the datasets generated. I consider the manuscript methodologically sound and suitable for publication in this journal

**********

what does this mean?). If published, this will include your full peer review and any attached files.

If you choose “no”, your identity will remain anonymous but your review may still be made public.

**Do you want your identity to be public for this peer review?**  For information about this choice, including consent withdrawal, please see our Privacy Policy

Reviewer #1: No

Reviewer #2: **Yes:**  Olmedo Jesus Cuaspud Caliz

**********

# Editor report: Acceptance letter

**DOI:** 10.1371/journal.pone.0359566.r004

**Authors:** Rongchun Han

PONE-D-26-17903R1

PLOS One

Dear Dr. Wang,

I'm pleased to inform you that your manuscript has been deemed suitable for publication in PLOS One. Congratulations! Your manuscript is now being handed over to our production team.

At this stage, our production department will prepare your paper for publication. This includes ensuring the following:

* All references, tables, and figures are properly cited

* All relevant supporting information is included in the manuscript submission,

* There are no issues that prevent the paper from being properly typeset

You will receive further instructions from the production team, including instructions on how to review your proof when it is ready. Please keep in mind that we are working through a large volume of accepted articles, so please give us a few days to review your paper and let you know the next and final steps.

Lastly, if your institution or institutions have a press office, please let them know about your upcoming paper now to help maximize its impact. If they'll be preparing press materials, please inform our press team within the next 48 hours. Your manuscript will remain under strict press embargo until 2 pm Eastern Time on the date of publication. For more information, please contact onepress@plos.org.

You will receive an invoice from PLOS for your publication fee after your manuscript has reached the completed accept phase. If you receive an email requesting payment before acceptance or for any other service, this may be a phishing scheme. Learn how to identify phishing emails and protect your accounts at https://explore.plos.org/phishing.

If we can help with anything else, please email us at customercare@plos.org.

Thank you for submitting your work to PLOS One and supporting open access.

Kind regards,

PLOS One Editorial Office Staff

on behalf of

Prof Dr Rongchun Han

Academic Editor

PLOS One
